# Supplementary material for: Protected areas network is not adequate to protect a critically endangered East Africa Chelonian: Modelling distribution of pancake tortoise, Malacochersus tornieri under current and future climates
Source: PLoS One. 2021 Jan 20;16(1):e0238669. doi: 10.1371/journal.pone.0238669 (PMC7816999; doi:10.1371/journal.pone.0238669)
Supplement: S1 Fig — Response curves were fitted through locally estimated scatterplot smoothing (LOESS). Grey background shows a scale from y-axis which is replicated to every graph. (DOCX) [file pone.0238669.s001.docx]

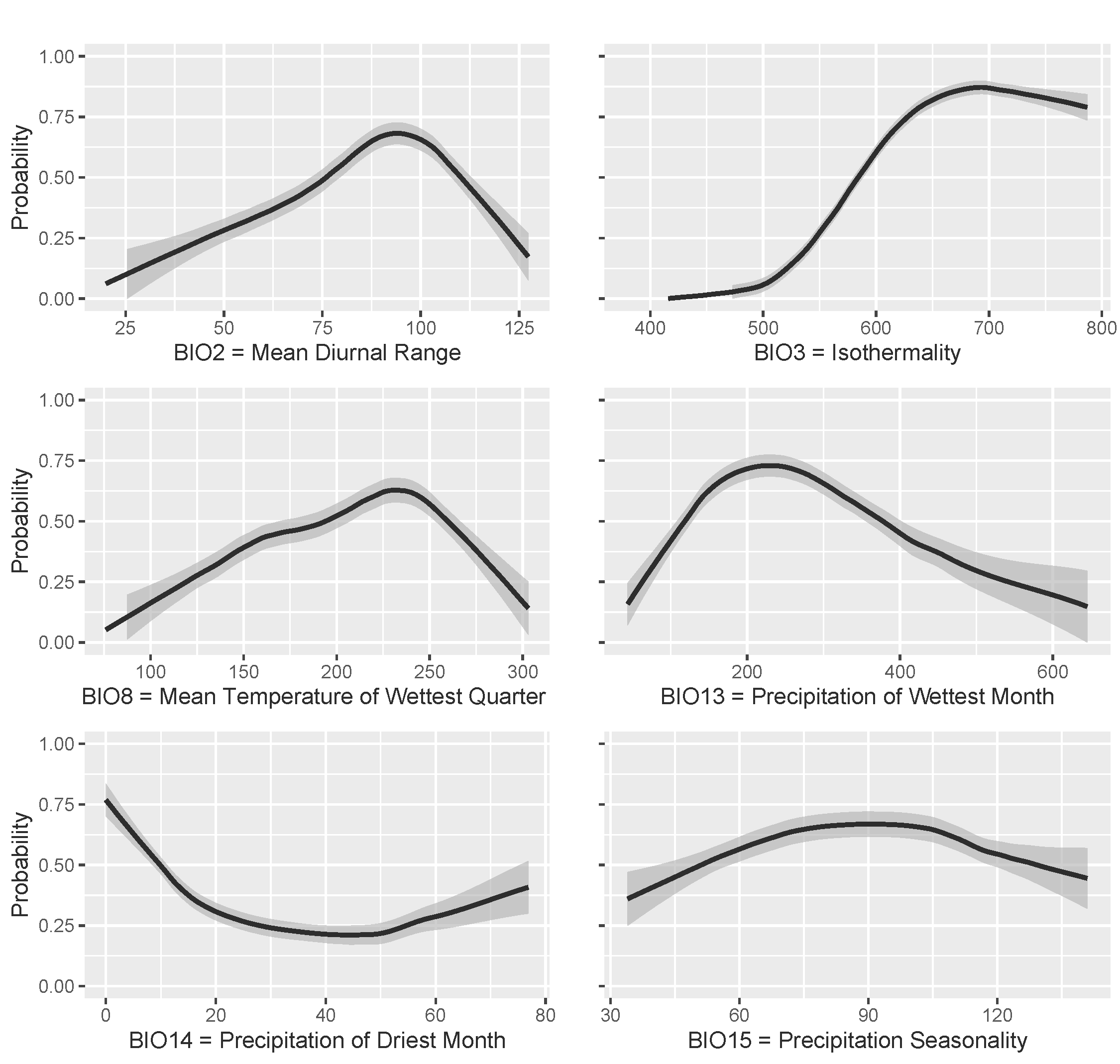


**S1 Fig:** Response curves from the ensemble models to the six selected bioclimatic variables. Response curves were fitted through locally estimated scatterplot smoothing (LOESS). Grey background shows a scale from y-axis which is replicated to every graph.
