# Supplementary material for: Protected areas network is not adequate to protect a critically endangered East Africa Chelonian: Modelling distribution of pancake tortoise, Malacochersus tornieri under current and future climates
Source: PLoS One. 2021 Jan 20;16(1):e0238669. doi: 10.1371/journal.pone.0238669 (PMC7816999; doi:10.1371/journal.pone.0238669)
Supplement: S2 Fig — BIO 8 = mean temperature of the wettest quarter, BIO 3 = the isothermality, BIO 2 = mean diurnal range, BIO 15 = precipitation seasonality, BIO 14 = precipitation of the driest month and BIO 13 = precipitation of the wettest month. (DOCX) [file pone.0238669.s002.docx]

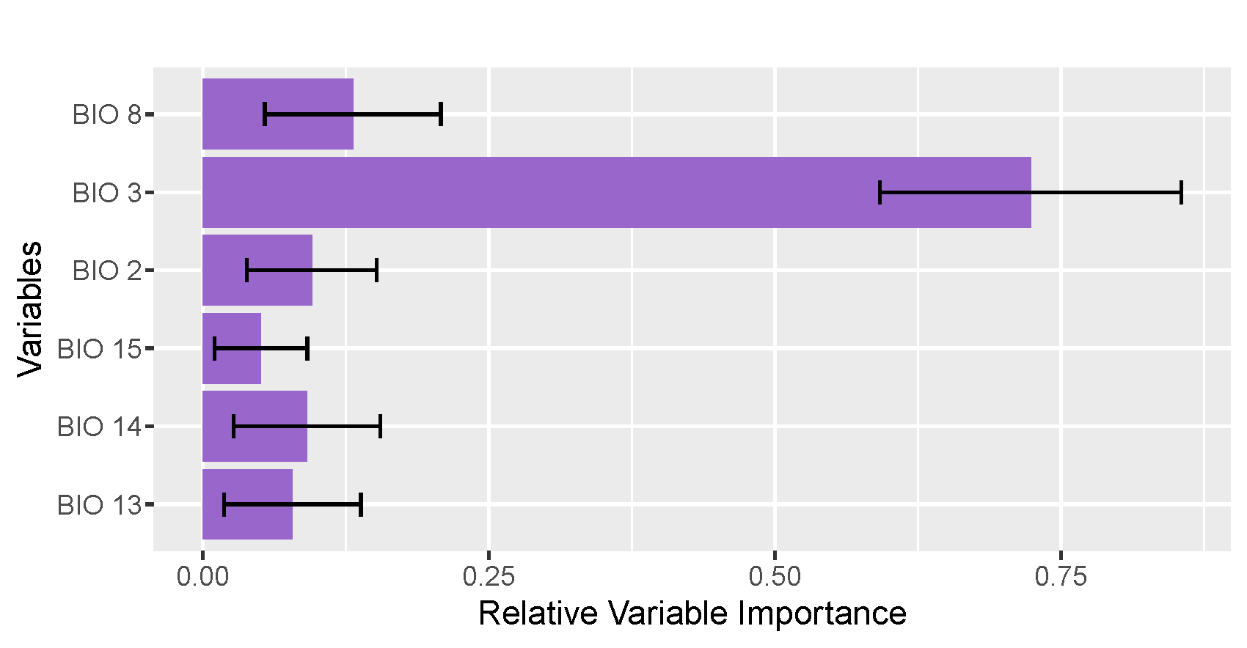


**S2 Fig:** Variable importance for six less correlated climatic variables of the ensemble species distribution model. BIO 8 = mean temperature of the wettest quarter, BIO 3 = the isothermality, BIO 2 = mean diurnal range, BIO 15 = precipitation seasonality, BIO 14 = precipitation of the driest month and BIO 13 = precipitation of the wettest month.
