# Supplementary material for: Protected areas network is not adequate to protect a critically endangered East Africa Chelonian: Modelling distribution of pancake tortoise, Malacochersus tornieri under current and future climates
Source: PLoS One. 2021 Jan 20;16(1):e0238669. doi: 10.1371/journal.pone.0238669 (PMC7816999; doi:10.1371/journal.pone.0238669)
Supplement: S1 Table — (DOCX) [file pone.0238669.s003.docx]

**S1 Table: Ten Global Circulation Models (GCMs) used in our study**

| **S/N** | **Acronym** | **Model** |
| --- | --- | --- |
| 1 | MIROC5 | Model for Interdisciplinary Research On Climate - Version 5 |
| 2 | CESM1-CAM5 | Community Earth System Model version 1 - Community Atmospheric Model - Version 5 |
| 3 | IPSL-CM5A-MR | Institute Pierre Simon Laplace - Version 5.A - Mid-resolution |
| 4 | FIO-ESM | First Institute of Oceanography Earth System Model |
| 5 | GISS-E2-H | Goddard Institute for Space Studies - Version. E2 - HYCOM Ocean |
| 6 | CSIRO-Mk3-6-0 | Commonwealth Scientific and Industrial Research Organization - Version Mk 3.6.0 |
| 7 | GISS-E2-R | Goddard Institute for Space Studies - Version. E2 - Russell Ocean |
| 8 | GFDL-ESM2G | Geophysical Fluid Dynamics Laboratory - Earth System Model - Version 2 - Generalized Ocean Layer Dynamics |
| 9 | MIROC-ESM-CHEM | Model for Interdisciplinary Research On Climate - Earth System Model - Atmospheric Chemistry Coupled Version |
| 10 | MRI-CGCM3 | Meteorological Research Institute - Coupled Global Climate Model - Version 3 |
