# Supplementary material for: Protected areas network is not adequate to protect a critically endangered East Africa Chelonian: Modelling distribution of pancake tortoise, Malacochersus tornieri under current and future climates
Source: PLoS One. 2021 Jan 20;16(1):e0238669. doi: 10.1371/journal.pone.0238669 (PMC7816999; doi:10.1371/journal.pone.0238669)
Supplement: Attachment — Submitted filename: Merow et al. 2013 Maxent.pdf [file pone.0238669.s004.pdf]

Forum

EDITOR'S CHOICE

Ecography 36: 1058–1069, 2013
doi: 10.1111/j.1600-0587.2013.07872.x
© 2013 The Authors. Ecography © 2013 Nordic Society Oikos
Subject Editor: Niklaus E. Zimmermann. Accepted 27 March 2013

A practical guide to MaxEnt for modeling species' distributions: what it does, and why inputs and settings matter

Cory Merow, Matthew J. Smith and John A. Silander, Jr

C. Merow (cory.merow@gmail.com) and J. A. Silander, Jr, Univ. of Connecticut, Ecology and Evolutionary Biology, 75 North Eagleville Rd., Storrs, CT 06269, USA. – M. J. Smith and CM, Computational Ecology and Environmental Science Group, Computational Science Laboratory, Microsoft Research Ltd., 21 Station Road, Cambridge, CB1 2FB, UK.

The MaxEnt software package is one of the most popular tools for species distribution and environmental niche modeling, with over 1000 published applications since 2006. Its popularity is likely for two reasons: 1) MaxEnt typically outperforms other methods based on predictive accuracy and 2) the software is particularly easy to use. MaxEnt users must make a number of decisions about how they should select their input data and choose from a wide variety of settings in the software package to build models from these data. The underlying basis for making these decisions is unclear in many studies, and default settings are apparently chosen, even though alternative settings are often more appropriate. In this paper, we provide a detailed explanation of how MaxEnt works and a prospectus on modeling options to enable users to make informed decisions when preparing data, choosing settings and interpreting output. We explain how the choice of background samples reflects prior assumptions, how nonlinear functions of environmental variables (features) are created and selected, how to account for environmentally biased sampling, the interpretation of the various types of model output and the challenges for model evaluation. We demonstrate MaxEnt's calculations using both simplified simulated data and occurrence data from South Africa on species of the flowering plant family Proteaceae. Throughout, we show how MaxEnt's outputs vary in response to different settings to highlight the need for making biologically motivated modeling decisions.

The MaxEnt software package (Phillips et al. 2006) is particularly popular in species distribution/environmental niche modeling, with over 1000 applications published since 2006. MaxEnt users are confronted with a wide variety of modeling decisions, from which input datasets to choose to multiple settings available in the software package. Guidance on the implications of different MaxEnt modeling decisions and their biological justifications are lacking, with the notable exceptions of Elith et al. (2010, 2011). The default settings seem often chosen as a consequence of unfamiliarity with the maximum entropy modeling method, even though alternatives are often more appropriate. As MaxEnt is used to address increasingly complex problems (and not simple exploratory analyses), it is important to ensure that modeling decisions are biologically motivated by specific hypotheses, study goals, and species-specific considerations and reflect the intended a priori assumptions (Peterson et al. 2011, Araujo and Peterson 2012).

Here, we provide a detailed explanation of the mechanics of MaxEnt, and a prospectus on modeling options, so that users can make informed decisions about choosing settings, inputs and outputs. In particular, we provide guidance on choosing background data, the range of functional forms of environmental variables (i.e. features) permitted, the degree to which MaxEnt regulates model complexity, controlling for sampling bias, interpreting different types of output and evaluating models (see glossary in Supplementary material Appendix 1).

II. How MaxEnt works

MaxEnt takes a list of species presence locations as input, often called presence-only (PO) data, as well as a set of environmental predictors (e.g. precipitation, temperature) across a user-defined landscape that is divided into grid cells. From this landscape, MaxEnt extracts a sample of background locations that it contrasts against the presence locations (section III.A). Presence is unknown at background locations.

Originally, MaxEnt was employed to estimate the density of presences across the landscape (Phillips et al. 2006). Density estimation implicitly assumes that individuals have been sampled randomly across landscape; i.e. samples occur in proportion to population density. When the total population size is known, such models predict the occurrence rate in a cell, defined as the expected number of individuals in that cell (Fithian and Hastie 2012). However, population size is typically unknown, so only relative comparisons among these rates are meaningful, resulting in a

relative occurrence rate (ROR; Fithian and Hastie 2012). Given that an individual was observed, the ROR describes the relative probability that the individual derived from each cell on the landscape. In other words, the ROR is the relative probability that a cell is contained in a collection of presence samples. The ROR corresponds to Maxent's raw output.

In contrast, species distribution modelers sometimes assume that grid cells (rather than individuals) on the landscape have been sampled randomly for presence. This naturally leads to models that predict probability of presence in each cell (Royle et al. 2012). These two sampling assumptions (individuals vs grid cells) can lead to similar results when presences are spatially sparse but are incompatible when multiple counts are likely to occur per grid cell (Renner and Warton 2012). MaxEnt can be used to predict probability of presence only by using a transformation of the ROR, called logistic output (Phillips and Dudik 2008), which relies on strong assumptions that have been criticized (section III.E; Royle et al. 2012).

Thus Maxent users have a dilemma: they can either assume PO data is a random sample of individuals (a questionable assumption) and predict RORs (a reasonable interpretation of MaxEnt's output) or they can assume the data represent a random sample of space (a reasonable assumption if sampling bias is not a problem) and predict probability of presence (a questionable interpretation of MaxEnt's output). If one is willing to forego the rigorous assumptions about sampling and the probabilistic interpretation of model output, it is still possible to simply interpret MaxEnt's predictions as indices of habitat suitability, which might be useful for qualitative exploratory analyses. Here, we make general observations that relate to any of these interpretations, but focus on predicted RORs to describe model specification in the next section because these are the fundamental quantities predicted by maximum entropy models.

MaxEnt predicts RORs as a function of the environmental predictors at that location. These RORs ($P^*(\mathbf{z}(x_i))$) take the form,

$$P^*(\mathbf{z}(x_i)) = \exp(\mathbf{z}(x_i)\boldsymbol{\lambda})/\Sigma_i \exp(\mathbf{z}(x_i)\boldsymbol{\lambda}) \quad (1)$$

where $\mathbf{z}$ is a vector of J environmental variables at location x_i, and $\boldsymbol{\lambda}$ is a vector of regression coefficients, with $\mathbf{z}(x_i)\ \boldsymbol{\lambda} = z_1(x_i)^*\lambda_1 + z_2(x_i)^*\lambda_2 + \ldots + z_J(x_i)^*\lambda_J$. These RORs sum to unity across the landscape because the denominator is a sum of the RORs over all grid cells in the study (called normalization). Normalization ensures that the occurrence rates are in fact relative occurrence rates.

A. Different derivations of MaxEnt's model

Equation (1) is part of a general class of models designed to predict RORs, which leads us to describe MaxEnt models from four related perspectives. Together, these descriptions provide a foundation for understanding MaxEnt models from both statistical and machine learning perspectives. We then proceed to describe the specific algorithm that MaxEnt uses to obtain a model.

1. A normalized Poisson model

Normalized exponential functions (Eq. 1) are a general class models that predict count data (i.e. the number of individuals; Warton and Shepherd 2010, Aarts et al. 2012, Fithian and Hastie 2012, Renner and Warton 2012, Royle et al. 2012). When the aim is to predict counts of occurrences, it is helpful to think of MaxEnt as a Poisson model where the number of counts is a function of environmental variables. Such models are commonly referred to as the resource selection functions in the habitat suitability literature (Manly et al. 2002, Keating and Cherry 2004, Johnson et al. 2006). This log-linear model has the form:

$$y_i \sim \exp(\mathbf{z}(x_i)\boldsymbol{\lambda}) \quad (2)$$

where y_i is the number of observations in cell i (Cressie 1993, Aarts et al. 2012, Fithian and Hastie 2012). Equation (2) is the occurrence rate in the numerator of Eq. 1, but lacks the normalization term in the denominator. Fithian and Hastie (2012) argue that predicting ROR is the best one can often do with typical PO data in a Poisson model. PO data rarely correspond to a unique record for each observed individual, and so if one were to obtain twice as many records in a particular region, it would be difficult to determine if that doubling were due to the occurrence of twice as many individuals or twice as much sampling. A number of studies have recently shown that the count model in Eq. (2) is a discretized approximation to more general class of inhomogeneous Poisson point process models that treat the landscape as continuous (Warton and Shepherd 2010, Chakraborty et al. 2011, Fithian and Hastie 2012, Renner and Warton 2012). Furthermore, Renner and Warton (2012) note that the assumption of spatial independence of PO samples may be frequently be violated, but that inhomogeneous Poisson point process models can remedy this to some extent.

2. A multinomial model

The counts observed over a landscape of N cells can equivalently be interpreted as a multinomial distribution, where

$$\{y_1, y_2, \ldots, y_N\} \sim \text{Multinomial}(P^*(\mathbf{z}(x_1)), P^*(\mathbf{z}(x_2)), \ldots, P^*(\mathbf{z}(x_N))) \quad (3)$$

(Dudik and Phillips 2009, He 2010). By using a multinomial model, the RORs automatically sum to unity across the landscape. Such models are referred to as density estimation because one is estimating the density of observations (presences) across the landscape (Fithian and Hastie 2012, Royle et al. 2012).

3. Maximizing entropy in geographical space

Equation (1) can be derived as the distribution with maximum entropy in geographic space (cf. Aarts et al. 2012). In other words, predictions are made for each cell on the landscape. The principle of maximum entropy postulates that models should be chosen that are as similar as possible to prior expectations while also being consistent with the data (Jaynes 2003, Dudik et al. 2004). The prior distribution, $Q(x_i)$, reflects the user's expectation about the distribution before accounting for the data. The relative entropy

(or Kullback–Leibler divergence) measures the similarity of the prediction to the prior by (Phillips and Dudik 2008):

$$\sum_{i=1}^{N} P^*(\mathbf{z}(x_i)) * log\left[\frac{P^*(\mathbf{z}(x_i))}{Q(x_i)}\right] \tag{4}$$

Usually the prior is a uniform distribution in geographic space, signifying that all cells are a priori equally likely to contain an individual (although other priors are possible; section III.D). This assumption corresponds to $Q(x_i) = 1/N$ (Eq. (4) then reduces to the Shannon entropy).

To ensure that the predictions are consistent with data, MaxEnt constrains the moments of the prediction (e.g. mean, variance) to match the empirical moments of the data. For example, one can constrain the prediction to have the same mean value of minimum July temperature, mean annual precipitation, etc., as the presence locations. For predictor *j*, with value z_j, the constraints can be written as:

$$\sum_{i=1}^{N} \mathbf{z}_{ij} P^*(\mathbf{z}(x_i)) = \frac{1}{M}\sum_{m=1}^{M} \mathbf{z}_{mj} \tag{5}$$

The left hand side of Eq. (5) describes the average value of z_j over the prediction while the right hand side describes the average value of z_j over the set of *M* presence locations. Many different distributions of $P^*(\mathbf{z}(x_i))$ might satisfy Eq. (5), so the maximum entropy principle selects the model that is most similar to the prior.

Maximizing the similarity between the prediction and the prior (section II.B) results in more general version of Eq. (1) that includes the prior distribution:

$$P^*(\mathbf{z}(x_i)) = Q(x_i)\exp(\mathbf{z}(x_i)\boldsymbol{\lambda})/\Sigma_i\, Q(x_i)\exp(\mathbf{z}(x_i)\boldsymbol{\lambda}) \tag{6}$$

where the sum in the denominator is over all grid cells in the study. Interpreting models in geographic space is helpful for understanding how spatially explicit models of sampling effort, which are incorporated in the prior distribution, affect ROR predictions (section III.D).

4. Minimizing relative entropy in environmental space

MaxEnt's predictions can be equivalently interpreted for a set of environmental conditions, i.e. in environmental space, irrespective of their spatial location (Elith et al. 2011, Aarts et al. 2012). This provides a particularly intuitive graphical interpretation of MaxEnt models (Fig. 1). Predictions in environmental space rely on comparing empirical probability densities of predictors. For a predictor Z, a probability density over all locations describes the relative likelihood of Z taking on different values across the landscape, written as P(z) (Fig. 1). Similarly, P(**z**) is a multivariate probability density over the vector of predictors **Z**. Note that P(**z**) is the probability associated with a set of predictors, whereas the notation for the previous three methods required $P(\mathbf{z}(x_i))$, the probability associated with a particular location x_i.

To understand MaxEnt's predictions in environmental space, three probability densities of **Z** are needed: the prior probability density, Q(**z**); the probability density of **Z** at presence locations, P(**z**); and the predicted ROR at each location in the landscape P*(**z**) (Fig. 1). In environmental space, enforcing the null hypothesis that the species is equally likely to be anywhere in the landscape corresponds to assuming that environment **Z** is used in proportion to its frequency. Thus we equate Q(**z**) with the multivariate probability density of predictors across the entire landscape (Elith et al. 2011). The observed density of predictors at presence locations, P(**z**), is then predicted by P*(**z**). In environmental

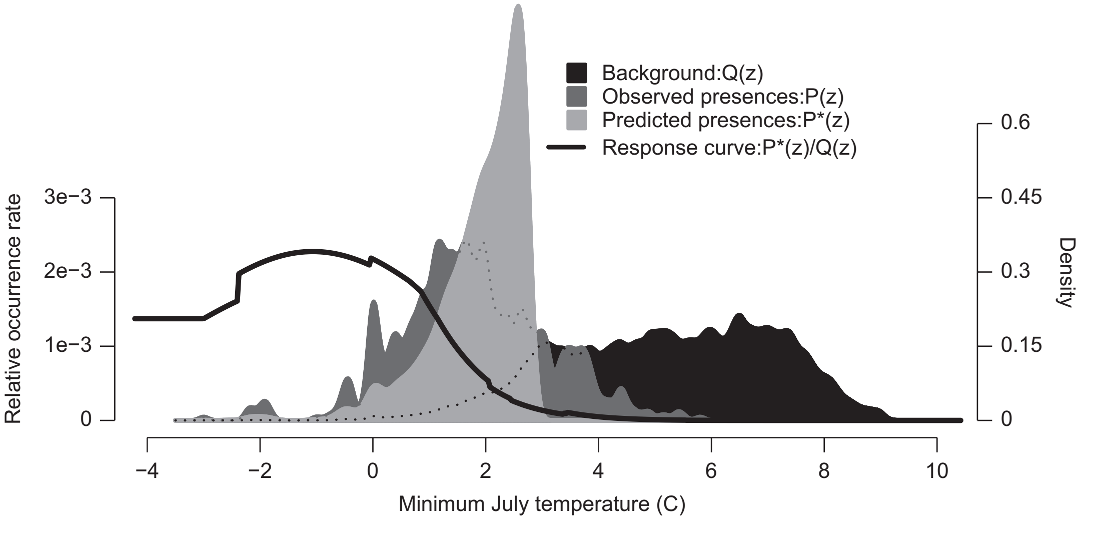

Figure 1. Illustration of calculating response curves for *P. punctata* in MaxEnt (with default settings) from probability densities of minimum July temperature (MJT). MaxEnt builds a model for the ratio of the probability density of MJT at presence locations (dark grey) to the probability density of MJT at background locations (black), denoted by P(**z**)/Q(**z**) (Eq. 8). This model, P*(**z**), is represented by the response curve (black line), a smoothed estimate of the actual ratio of these densities. The response curve tends to pick up jumps in the ratio (e.g. near x = 0, 2 and 3.2), as well as broad, smoothed trends (e.g. the unimodal shape). The sharp spikes in the response curve near x = 0 result from using two threshold features. Clamping is illustrated at the left edge of the response curve. The light grey probability density represents predicted presences based on thresholded model output (for illustration).

space, MaxEnt maximizes the similarity between P*(**z**) and Q(**z**), and predicts the ROR in environment **z** as:

$$P^*(\mathbf{z}) = Q(\mathbf{z})\exp(\mathbf{z}\boldsymbol{\lambda})/\Sigma_i\, Q(\mathbf{z})\exp(\mathbf{z}\boldsymbol{\lambda}) \quad (7)$$

Here, the sum in the denominator is defined over the set of distinct predictors (**Z**), as opposed to the set of spatial locations in Eq. (6), although these are equivalent formulations. Conveniently, Eq. (7) can then be rearranged to illustrate how MaxEnt models the ratio of P(**z**) to Q(**z**) shown in Fig. 1:

$$P(\mathbf{z})/Q(\mathbf{z}) \approx P^*(\mathbf{z})/Q(\mathbf{z}) = \exp(\mathbf{z}\boldsymbol{\lambda})/\text{constant} \quad (8)$$

High values of P*(**z**) are observed when P(**z**) is large relative to Q(**z**) (Fig. 1). See Fig. 1 for an illustration of how MaxEnt maximizes the similarity of the prediction to the prior: the probability density of the minimum July temperature at predicted presences (light grey) has a similar mean to the density from observed presences (dark grey), however, the mode of predicted presences is shifted towards the mode of the background (black), compared to the mode of the observed presences). This occurs because minimizing the relative entropy of the predicted distribution with respect to the prior makes it as similar as possible to the density of background locations while still satisfying constraints imposed by the density of presence locations (e.g. similar means).

B. The MaxEnt implementation

Here, we discuss the means by which the MaxEnt software package makes predictions (see Supplementary material for an illustration of MaxEnt's calculations in an Excel worksheet). MaxEnt's treatment of the predictors derives from the field of machine learning. In most statistical models, **Z** represents a handful of predictors, e.g. temperature, precipitation, etc., selected a priori by the user. In contrast, MaxEnt derives a number of so-called features for each predictor, each of which is a simple mathematical transformation of the predictor (linear, quadratic, product, threshold, hinge; section III.B.). Here, we use the term predictor to refer to the environmental covariates themselves and features to refer to the mathematical transformations of these predictors created by MaxEnt (sometimes called a basis expansion). The role of the features is depicted by response curves, which plot predicted ROR against the values of a particular predictor (Fig. 1, 2) and provide an important tool for evaluating the biological plausibility of the model. The user can choose which types of features to use and obtain either the complex, highly nonlinear response curves typical of MaxEnt, or the simpler response curves composed of fewer features typical of statistical models.

To obtain a solution, MaxEnt maximizes the so-called gain function, a penalized maximum likelihood function. Exponentiating the gain function gives the likelihood ratio of an average presence to an average background point, so maximizing the gain corresponds to finding a model that can best differentiate presences from background locations. Using the formulation in geographic space, one can substitute Eq. 6 into Eq. 4 to obtain the first two terms in the gain function, while the third term is a penalty to reduce overfitting (Dudik et al. 2004, Phillips et al. 2006):

$$gain = \underbrace{\frac{1}{m}\sum_{i=1}^{M} \mathrm{z}(\mathrm{x}_i)\boldsymbol{\lambda}}_{\substack{\text{sum of predicted}\\ \text{values at}\\ \text{presence locations}}} - log \underbrace{\sum_{i=1}^{N} Q(\mathrm{x}_i)\, e^{\mathbf{z}(\mathrm{x}_i)\boldsymbol{\lambda}}}_{\substack{\text{sum of predicted}\\ \text{values at}\\ \text{background locations}}} - \underbrace{\sum_{j=1}^{J} |\lambda_j| * \beta * \sqrt{s^2[z_j]/M}}_{\substack{\text{overfitting}\\ \text{penalty}}} \quad (9)$$

where β is a regularization coefficient, and $s^2[z_j]$ is the variance of feature j at presence locations. The first term describes the likelihood of the presence data; Eq. (4) shows that the predicted ROR increases with the value of $\mathbf{z}(x_i)\boldsymbol{\lambda}$, so presence locations should be assigned large values of $\mathbf{z}(x_i)\boldsymbol{\lambda}$ to increase the gain. The second term describes the likelihood at all N background locations (section III.A). Since this term is negative, the gain is reduced if large values of $\mathbf{z}(x_i)\boldsymbol{\lambda}$ are assigned to background locations. The choice of landscape, and how it is discretized, will clearly affect predictions (section III.A). Embedded in the second term is the prior distribution $Q(z)$, which down weights the importance of sites that are expected to contain the species (the predictors **z** can only describe how the observed occurrence pattern differs from our expectations). Thus prior assumptions about the species distribution, or the sampling of it, clearly affect predictions (section III.D). The third term in Eq. (9) is a regularization, or LASSO, penalty, and is used in statistics and machine learning to reduce model overfitting (Tibshirani 1996, Hastie et al. 2009). Regularization forces many coefficients to be zero and retains only those that improve the first two terms in Eq. (9) enough to offset the penalty in the third term. The regularization coefficient, β in Eq. (9), must be defined by the user, and determines the strength of the penalty. The regularization penalty is proportional to variance of the feature j at presence locations, $s^2[z_j]$, based on the rationale that features with larger variance should incur a larger penalty and be less likely to be included in the model (Phillips and Dudik 2008). The regularization penalty is inversely proportional to the square root of the sample size, which reduces the effect of regularization as sample size increases.

III. Model settings: how they work and how to choose them

We have identified six key decisions about input data and settings that can critically influence the models fitted by

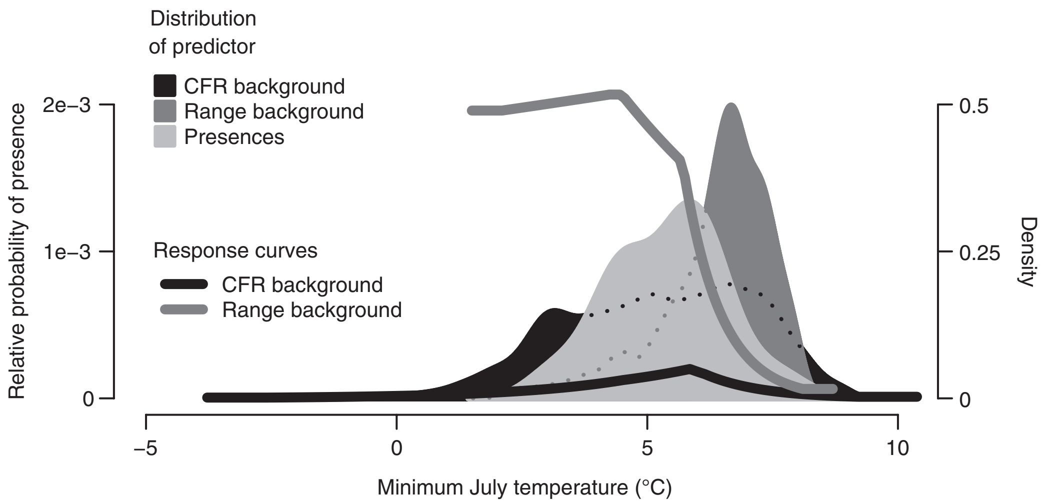

Figure 2. Illustration of the variability in response curves derived from different background samples for *P. lacticolor*. Filled densities represent the distribution of minimum July temperature (MJT) values in the entire Cape Floristic Region (black), a region encompassing just the species known range (dark gray), and at sampled presence locations (light grey). The black and dark grey distributions represent different ways of modeling Q(**z**) in Eq. 7 based on background samples drawn from different spatial extents. Solid lines represent response curves fit by MaxEnt using default features classes and only MJT as a predictor (black: background from Cape Floristic Region; dark grey: background from known range).

MaxEnt. For each of these, we first describe how the decisions influence the relevant MaxEnt calculations and then discuss general ecological and computational considerations for making these choices. Our goal is to provide users with the necessary understanding to make their own modeling decisions, which should be specific to species biology, study goals and data limitations. Detailed demonstrations and related technical details are contained in Supplementary material appendices paired with each subsection.

For illustrative purposes, we model the range limits of *Protea punctata* (692 presences) and *Protea lacticolor* (27 presences), two woody shrubs inhabiting Mediterranean climate fynbos shrubland communities in the Cape Floristic Region (CFR) of South Africa. Samples were obtained as part of the Protea Atlas Project (Rebelo 2002), an exhaustive survey of the Proteaceae family across the CFR (> 250 000 records over 90 000 km^2). Importantly, the samples span the complete extent of the species' ranges and cover all fynbos habitats where any species of Proteaceae might occur. Knowledge of the sampling locations for the atlas greatly facilitates the assessment of sampling bias, which appears to be small (Supplementary material Appendix 5, Fig. E1). Low sampling bias may be atypical for biological occurrence records but in our case allows for an ideal data set for illustration. For modeling, we used a set of 24 environmental variables (Supplementary material Appendix 2) suggested by Latimer et al. (2006) for the Proteaceae that includes both climatic (Schulze 1997) and edaphic variables. These are at 1 arc minute spatial resolution (approximately 1.56 × 1.85 km), enabling us to characterize the high topographic and edaphic variation across the CFR (Linder 2005).

A. Background data

How it works

MaxEnt contrasts presences against background locations where presence/absence is unmeasured (Fig. 1; Eq. (9); Phillips and Dudik 2008). Confusingly, background samples are sometimes called pseudoabsences (Phillips et al. 2009). Absences are desired because they allow one to predict probability of presence (e.g. using logistic regression; Peterson et al. 2011, Anderson 2012) and without absences predictions are typically confined to ROR (Ward et al. 2009; but see section III.E).

By default, MaxEnt uses a prior, Q(x), which assumes that the species is equally likely to be in anywhere on the landscape. This assumes that every pixel x has the same probability of being selected as background (in geographic space), or equivalently that every environment **z** has a probability of being selected as background according to its frequency P(**z**) (in environmental space). Modifying the background sample is therefore equivalent to modifying the prior expectations for the species' distribution. Note that by using a uniform prior, MaxEnt predicts a distribution that is as spatially diffuse as possible, which tends to predict the largest possible range size consistent with the data.

To see how different background samples affect response curves, consider the relationship between the ROR for *P. lacticolor* and the minimum July temperature (MJT) gradient for different background choices in Fig. 2. When background is selected from a region larger than *P. lacticolor*'s range, such as the Cape Floristic Region, the MJT gradient spans locations with both higher and lower MJT than where *P. lacticolor* is observed and produces a unimodal response curve. When background is selected only from a smaller region encompassing just *P. lacticolor*'s known range, a (roughly) monotonic response curve is obtained because values of MJT lower than *P. lacticolor*'s tolerance are not included in the background sample. Hence, the choice of background sample can alter the features selected by MaxEnt based on the range of the environmental gradients it spans. Neither response curve is more correct than the other; this highlights the need for an ecological justification for background selection. Understanding the impact of the

background on response curves is particularly critical when extrapolating to novel environmental scenarios (Elith et al. 2010, Webber et al. 2011).

Conceptually, MaxEnt contrasts the environmental conditions at the background locations with those at observed presence locations (using the ratio $P(\mathbf{z})/Q(\mathbf{z})$; Fig. 1). For example, consider fitting a model to MJT data only, which ranges from 2 to 10°C across some hypothetical region. If 75% of presences are found in locations with MJT > 8°C, one might be tempted to conclude that the species prefers warmer locations. But if 95% of the landscape consists of locations with MJT > 8°C, one should actually arrive at the opposite conclusion: the species prefers the lower MJT locations, but high MJT locations are primarily available. Thus MaxEnt's conclusions depend on whether MJT is uniformly distributed over the background or skewed (Fig. 2).

How to choose

We recommend that the background sample should be chosen to reflect the environmental conditions that one is interested in contrasting against presences based on the spatial scale of the ecological questions of interest (Saupe et al. 2012). Often, this contrast is made against locations that are accessible via dispersal. If one uses the default settings for background selection (a uniform prior in geographic space), the background extent should contain only locations where the species is equally likely to reach.

A number of studies have documented the variability in predictions that can emerge from different background samples for MaxEnt, with a particular focus on the extent of the region from which they are selected (Fig. 2; Phillips et al. 2009, VanDerWal et al. 2009, Anderson and Raza 2010, Baasch et al. 2010, Elith et al. 2010, 2011, Giovanelli et al. 2010, Yates et al. 2010, Anderson and Gonzalez 2011, Barve et al. 2011, Saupe et al. 2012). There is considerable flexibility for users to specify exactly which points to use as background, choosing their total number, or the spatial extent from which they are chosen. For example, if one were interested in determining the best location for a reserve for *P. punctata* then the background should be drawn only from the species known range, whereas if one were interested in which Mediterranean-climate regions it might invade worldwide in the absence of dispersal limitation (hypothetically) then the background should be chosen from these habitats across the globe (Barve et al. 2011, Webber et al. 2011; more examples in Supplementary material Appendix 3).

B. Features

How it works

MaxEnt is capable of building very complex, highly nonlinear response curves (Fig. 1, 2) using a variety of feature classes. For example, if we use precipitation as a predictor, the linear feature class ensures that the mean value of precipitation at where the species is predicted to occur approximately matches the mean value where it is observed to occur (Eq. 5). A quadratic feature constrains the variance in rainfall where the species is predicted to occur to match observation. A product feature constrains the covariance of rainfall with other predictors and is equivalent to interaction terms in regression (when linear features are also included). Threshold features make a continuous predictor binary by generating a feature whose value is 0 below the threshold and 1 above. Hinge features are like threshold features, except that a linear function is used, instead of a step function (Supplementary material Appendix 1; Phillips and Dudik 2008). Categorical features (e.g. land-use) split a predictor with *n* categories into *n* binary features, which take the value 1 when the feature is present and 0 otherwise. All features are rescaled to the interval [0,1] to make the coefficients comparable.

By default, MaxEnt uses the number of presences to determine which feature classes to use; more presences allows more features and > 80 presences leads to all feature classes being used. However, the user can also specify the feature classes manually. Consider a model with 19 predictors, which might occur if one selects the 'Bioclim' predictors from Worldclim (Supplementary material Appendix 4, Table D2; Hijmans et al. 2005). One linear and quadratic feature is constructed for each predictor. Product features are constructed for each pair of predictors, giving a total of $19!/2!17! = 171$ unique product features. The number of possible piecewise (threshold and hinge) features depends on the number of presences. MaxEnt permits a threshold, forward hinge, and backward hinge (Supplementary material Appendix 1) feature between each pair of successive values of a predictor. For example, if there are 100 presence observations the number of piecewise features is given by: 3 (types of piecewise functions) × 99 (pairs of data point) × 19 (predictors) = 5643. This collection of features is explored by MaxEnt, and the most useful features are extracted. The features retained, along with their coefficients (λ) and minimum/maximum values of the feature, can be found in a file written to MaxEnt's output directory with file extension. 'lambdas'.

How to choose

We recommend that users minimize correlation among predictors and identify the appropriate feature shapes prior to model building (depending on study goal). From the collection of biologically plausible predictors, we recommend removing highly correlated predictors using correlation analysis, clustering algorithms, principal components analysis or some other dimension reduction method because the complex features created by MaxEnt are often already highly correlated. If projection or interpretation of the species' distribution or its environmental drivers is the goal, prescreening the predictors and their feature classes will lead to parsimonious and interpretable models. This approach corresponds to treating MaxEnt as a traditional statistical model (cf. Renner and Warton 2012). An alternative school of thought based in machine learning, suggests including all reasonable predictors in the model and letting the algorithm decide which ones are important, via regularization (section III.C; Phillips et al. 2006). Elith et al. (2011) have noted that high collinearity is less of a problem for machine learning methods compared to statistical methods, but we caution that this is only true if predictive accuracy of the presences is the study goal. The MaxEnt software package can accommodate either approach, however it uses the machine learning approach by default.

In considering feature selection, it is useful to think about species responses to environmental gradients (cf.

Austin 2002, 2007; Fig. 2). While Austin (2002) argues that species responses are often nonlinear, we note that including too much flexibility may make it challenging to differentiate noise from nonlinear signals in real data sets. Ecological theory suggests response curves are (at least for fundamental niches) often unimodal (Austin 2007) and hence quadratic features may be appropriate. However if a species' niche is truncated such that the one side of the unimodal curve is not part of the background sample (e.g. chilling responses not sampled for tropical species), a linear feature might be sufficient. In this sense, feature selection can be intertwined with the selection of the study region. Omitting product features may be desirable because the (marginalized) response curves for each predictor completely define the model and are easier to interpret than those that depend on the values of other predictors (so long as interactions are negligible). Threshold terms are appealing when a known physiological tolerance limit exists, such as a freezing tolerance threshold. Hinge features provide a generalization of linear and threshold features and might characterize processes that initiate at a threshold and increase linearly (e.g. stomate controlled transpiration, or enzyme induction). A model using only hinge features produces complex but smoothed response curves that are much like GAMs (Elith et al. 2010). Austin (2002) observes that empirical response curves are often skewed and that this may be due to competition; one can constrain the skew using a cubic term or multiple hinge features, but this must be done by manually constructing features outside the MaxEnt software (Supplementary material Appendix 4). Finally, we note that the generality of any feature can be evaluated with cross-validation (section III.F).

C. Regularization

How it works

While the user can specify the feature classes to be used a priori, MaxEnt selects individual features (for each predictor) that contribute most to model fit using regularization (Phillips et al. 2006). Regularization has been known to perform well in a variety of applications (Hastie et al. 2009) and is very efficient at selecting tens to hundreds of useful features from a candidate set of thousands to millions (Supplementary material Appendix 3, Table C1). Regularization is conceptually similar to the commonly used AIC and BIC diagnostics for model comparison (Burnham and Anderson 2002), in that it is based on a combination of likelihood and a complexity penalty (cf. Warren and Seifert 2011). Regularization can also be interpreted as placing a Bayesian prior distribution on the parameters (Goodman 2003).

Regularization reduces over-fitting in two ways. First, it ensures that the empirical constraints (Eq. 5) are not fit too precisely (Supplementary material Appendix 3, Fig. C2). One expects some imprecision in the empirically measured constraints, so it is better to require that predictions approximately satisfy constraints rather than to satisfy them exactly. Second, regularization penalizes the model in proportion to the magnitude of the coefficients, and therefore shrinks many coefficients toward zero while setting others to zero, thereby removing many features from the model (Tibshirani 1996).

The regularization coefficient, β in Eq. (9), is set by default for each feature class (linear, quadratic, etc.; Phillips and Dudik 2008) but can be tuned by multiplying it by a user-specified constant to amplify or dampen its effect to reflect the desired level of confidence and produce more or less complex models, respectively (Anderson and Gonzalez 2011, Elith et al. 2011). If it is important to ensure that a model is fit with all candidate features (e.g. to test a particular hypothesis), one can set the regularization coefficients to zero, but this should only be done when the number of features is small relative to the number of presences.

How to choose

We recommend exploring a range of regularization coefficient values and choosing a value that maximizes some measure of fit on a cross-validation data set (section III.F; Supplementary material Appendix 4, Fig. D1). The default regularization values have been chosen based on performance across a range of taxonomic groups (Phillips and Dudik 2008), and may be useful when building models for many species simultaneously, when species-specific tuning is impractical. However, many studies focus on just a few species, for which the default regularization coefficients may not be optimal (Phillips and Dudik 2008, Elith et al. 2010, Anderson and Gonzalez 2011).

Importantly, it may be possible to produce simpler models that have similar performance to more complex models by using a priori predictor and feature selection and increased regularization (Supplementary material Appendix 4, Fig. D3). Highly nonlinear response curves may also be undesirable because they may capture variation in correlated but unmeasured (i.e. latent) predictors rather than the species' response to the predictor of interest. Default regularization often retains hundreds of correlated features, so when biological interpretation is important, it may be more helpful to seek simpler models (see models that retain hundreds of features in Supplementary material Appendix 4, Table D1 and Fig. D3). Often, overly complex models will extract qualitatively similar distribution patterns and response curves to those of simpler models because excess complexity simply models noise, while the dominant patterns still persist (Warren and Seifert 2011, Syfert et al. 2013). However, coefficients often vary substantially between simple and complex models (Supplementary material Appendix 4, Table D1). While one can increase the regularization coefficients to remove more features, one should be cautious because it has the side effect of divorcing predicted values of constraints from empirical values (Supplementary material Appendix 4, Fig. D2). A suite of tools for evaluating whether competing models are significantly different from one another is available in ENMTools (Warren et al. 2010).

D. Sampling bias

How it works

By default, MaxEnt models are fit assuming that all locations on the landscape are equally likely to be sampled. However, occurrence data sets typically exhibit some sampling bias, wherein some environmental conditions (near towns, roads,

etc.) are more heavily sampled than others, particularly when samples derive from museum specimens (Reddy and Dávalos 2003, Graham et al. 2004, Phillips et al. 2009). The uniform sampling assumption does not require a uniformly random sample from geographic space, but instead that environmental conditions are sampled in proportion to their availability, regardless of their spatial pattern (i.e. a sample from P(**z**); Aarts et al. 2012).

When sampling is biased, one cannot differentiate whether species are observed in particular environments because those locations are preferable or because they receive the largest search effort (Phillips et al. 2009, Sastre and Lobo 2009, Wisz and Guisan 2009, Newbold et al. 2010, Chakraborty et al. 2011). For PO data, the probability that an individual was recorded at a location can be decomposed into the product of the probability of sampling the location, the probability of detecting an individual there, and the ROR (Yackulic et al. 2012). Typically, MaxEnt users either implicitly or explicitly assume that detection probability and sampling probability are constant across space and thus do not account for any sampling bias (Yackulic et al. 2012). For PO data, one must explicitly model the probability of sampling a location because no absence data exist to fully describe which locations were searched. Models for detection probability can be constructed from repeated sampling of the same locations (cf. Kery et al. 2010).

Accounting for sampling bias is similar to background selection in the sense that changing either reflects different prior expectations. By accounting for sampling bias, the null hypothesis states that individuals are uniformly distributed in geographic space and that the only reason they have been observed in particular locations is because those are the only places that were sampled. Thus the prior distribution for the species' occurrence is the sampling distribution.

Data sets with explicit information on search effort (e.g. Breeding Bird Survey), allow sampling bias models to be formulated in geographic space (Supplementary material Appendix 4). Since this search effort is often unknown, methods to account for sampling bias are typically based on Target Group Sampling (TGS; Ponder et al. 2001, Phillips et al. 2009). TGS uses the presence locations of taxonomically related species observed using the same techniques as the focal species (usually from the same database) to estimate sampling, under the assumption that those surveys would have recorded the focal species had it occurred there (Phillips et al. 2009).

Models of sampling effort or TGS locations can be incorporated into MaxEnt using one of two strategies: using a biased prior gives a nonuniform weighting to a given set of background points, while a biased background uses a uniform prior but modifies the selection of background points (Dudik et al. 2005, Phillips et al. 2009). For the biased prior method, the user provides an estimate of the relative search effort in each location on the landscape (the FactorBiasOut method described by Phillips et al. 2009). This is the most straightforward way to account for sampling bias in MaxEnt (Phillips et al. 2009). The biased prior has the same interpretation as the predicted ROR and reflects the assumption that the probability of observing an individual in a given location is based on the search effort there (details in Supplementary material Appendix 5, Table E1 and Appendix 6).

For the biased background approach (the DebiasAverages approach of Dudik et al. 2005) the user uses prior-information on the distribution of survey effort across a landscape to pre-select background locations before running MaxEnt. Biased background points are typically drawn only from TGS locations (Syfert et al. 2013). These locations are then passed to MaxEnt using the 'samples with data' format (see MaxEnt's tutorial). Unlike the biased prior method, this method does not directly incorporate any estimate of sampling effort into the MaxEnt training algorithm. This approach is motivated by the analogous case in presence–absence models, where the effect of sampling bias cancels out because it is common to presences and absences (Phillips et al. 2009). This assumption is challenging to evaluate for PO data precisely because ascertaining the importance of sampling bias is not possible when sampling effort is unknown.

How to choose

We recommend that users always attempt to account for sampling bias. Approximations of sampling bias should be preferentially be derived from direct sampling measures (e.g. survey locations from Breeding Bird Survey). If such data is not available then users could either build a biased prior by modelling the distribution of TGS samples using different covariates than those included in the occurrence model, or build a biased prior from the distribution of sampling effort across the TGS (simply, a grid of relative sample frequency). If it is not possible to approximate sampling effort using TGS species then users should attempt to pre-select background localities based on prior-knowledge of sampling effort. Users should always provide strong support for approximations of sampling effort that are inferred (e.g. through TGS; Syfert et al. 2013), rather than directly derived from standardized surveys. When it is difficult to evaluate whether the assumptions of TGS are fulfilled, models should only be used for exploratory purposes, as there is no substitute for data.

Using a biased prior is preferred if sampling probability can reasonably be estimated across the entire landscape (Supplementary material Appendix 5, Fig. E2, E3). A biased prior can be constructed using MaxEnt: TGS locations are provided to MaxEnt as if they represented a single species and the resulting prediction estimates sampling effort. Predictors might include distance to urban centers or roads, elevation, or topographic roughness (Phillips et al. 2009). Biased background sampling is necessary if sampling effort cannot be estimated across the entire landscape, but many TGS samples are available (Supplementary material Appendix 5, Fig. E2, E3). If very few TGS samples are available it will be very challenging to estimate sampling bias via either method, although this may represent the cases in which sampling bias is most prevalent. Presence locations are included in the background by MaxEnt, so using too few TGS locations will make it appear as though the species uses the environmental conditions in nearly the proportion in which they occur, which leads to dampened relationships to gradients and more spatially uniform predictions (Supplementary material Appendix 3, Fig. C3).

All options for discerning sampling bias from PO data rely on strong assumptions when sampling locations are

unknown. To determine if sampling bias is a problem, one can compare the distribution of sampled (TGS) locations to the distribution of background locations in environmental space (Supplementary material Appendix 5, Fig. E1). If these distributions are similar, then sampling bias is negligible for this choice of background. Alternatively, one could detect sampling bias by building a model with potentially biased samples and evaluating the predictions against a spatially independent data set (so long as both data sets do not contain the same bias; Anderson 2012, Syfert et al. 2013). Accurate predictions imply negligible sampling bias.

E. Types of output

How it works

MaxEnt produces three different types of output for its predictions: raw, cumulative and logistic. All three output types are related monotonically, so rank-based metrics for model fit (e.g. AUC) will be identical (Elith et al. 2011). However, the output types have different scaling that leads to different interpretations and to prediction maps that appear very different visually (Supplementary material Appendix 6, Fig. F2).

MaxEnt's raw output is interpreted as an ROR. The ROR sums to unity if all locations on the landscape are included in the background. Cumulative output assigns a location the sum of all raw values less than or equal to the raw value for that location and rescales this to lie between 0 and 100. Cumulative output can be interpreted in terms of an omission rate because thresholding at a value of *c* to predict a presence/absence surface will omit approximately *c*% of presences (Phillips and Dudik 2008). Logistic output, denoted L(**z**), uses transforms the raw output, as (Phillips and Dudik 2008):

$$L(\mathbf{z}) = \tau e^{\mathbf{z}\lambda - r}/(1 - \tau + \tau e^{\mathbf{z}\lambda - r}) \quad (10)$$

where *r* is the relative entropy of $P^*(\mathbf{z}(x_i))$ to $Q(\mathbf{z}(x_i))$ (Eq. 4). Phillips and Dudik (2008) propose that τ can be interpreted as the probability of presence at 'average' presence locations and that logistic output can be interpreted as probability of presence. MaxEnt does not fit a value of τ from the data, but arbitrarily assumes $\tau = 0.5$ as the default (Elith et al. 2011), which can have drastic consequences on the predicted probabilities assigned to each location (Supplementary material Appendix 6, Fig. F1; Royle et al. 2012).

How to choose

We recommend a) using raw output whenever possible, because it does not rely on post-processing assumptions; b) cumulative output when interpretations relate to omission rate (e.g. drawing range boundaries); c) avoiding logistic output because it is based on strong assumptions about the value of τ. Note that comparison or combining any output types among species is problematic unless the species have similar population density because species with similar spatial distributions may have very different prevalence (see Box 2 in Elith et al. (2011)).

Raw output is useful for comparing different models for the same species using the same background samples. Raw values depend on the number of locations in the landscape, making comparison challenging among models fit with different numbers of background sample points, spatial resolutions or extents (Phillips and Dudik 2008). Raw values can be skewed because they derive from an exponential model, and therefore may not be well calibrated with actual differences in suitability (Phillips and Elith 2010). Cumulative values can be problematic when small differences exist between a large subset of cells because these cells will be ranked from highest to lowest in spite of potentially negligible differences. Logistic outputs solve the problem of comparing models with different spatial scales at the expense of assumptions about the value of τ and may produce better calibrated models (Phillips and Dudik 2008, Phillips and Elith 2010).

If estimating probability of presence is necessary for a particular application, it may be reasonable to estimate τ or prevalence (or better, a conservative range of values) under circumstances where independent data are available, but the default value of τ is not appropriate without some biological justification (Supplementary material Appendix 6, Fig. F1). Lele and Keim (2006) and Royle et al. (2012) suggest a method for estimating prevalence from presence only data, however the estimates tend to have large variance except when using very large data sets.

F. Evaluating models

How it works

A full treatment of evaluating model predictions is outside the scope of this study (Peterson et al. 2011), however we discuss some standard diagnostics output by MaxEnt. One objective of model evaluation is assessing generality, in the sense that the model identifies attributes of the species distribution and not simply artifacts of a noisy sampling process. Generality can be obtained by penalizing models for complexity or using cross-validation. Penalizing for model complexity is done internally in MaxEnt using regularization (section III.C), but Warren and Seifert (2011) have suggested augmenting this by using AIC and BIC to compare competing MaxEnt models. MaxEnt provides a number of options for cross-validation, where presence locations are usually split into training data, used to fit the model, and test data, used to evaluate model predictions. K-fold cross-validation represents the most popular choice wherein the data are split into *k* independent subsets, and for each subset, the model is trained with $k-1$ subsets and evaluated on the kth subset.

To perform many types of model evaluation, metrics of model fit are needed (cf. Liu et al. 2010). Area under the receiver-operator curve (AUC) has emerged as the most popular in the MaxEnt literature. AUC is a threshold independent measure of predictive accuracy based only on the ranking of locations. AUC is interpreted as the probability that a randomly chosen presence location is ranked higher than a randomly chosen background point. Note that AUC is traditionally used to determine how the model distinguishes between presences and absences, but with PO data AUC compares presences with background points.

As an alternative to AUC, creating binary, presence–absence predictions is useful for fit metrics based on

confusion matrices (Liu et al. 2005) or displaying simple maps. Thresholding makes continuous output binary by choosing a value of the ROR below which a species is considered absent and above which it is considered present. MaxEnt provides a number of methods to choose the value of the threshold, including minimum predicted value at a presence location, equal sensitivity and specificity on training data, and arbitrary values with user-specified omission.

How to choose

We recommend evaluating models based on their predictive accuracy on statistically independent cross validation data sets using fit metrics based on sensitivity (correctly predicted presences) and avoiding thresholding whenever possible. Cross-validation may be preferable to penalty functions for assessing model generality because it can be challenging to determine the appropriate strength of the penalty. K-fold cross validation is appealing because it uses the data efficiently and enables one to report the range, standard error, etc., of any model fit metrics over the *k* folds. K-fold cross-validation simultaneously allows one to assess uncertainty in predictions, another focus of model evaluation. Disadvantages of using cross validation, are that only part of the data can be used for model fitting and that it is challenging to obtain test data that is statistically (spatially) independent of training data (but see Hijmans 2012, Wenger and Olden 2012). Spatially correlated folds can lead to overestimates of model performance and underestimates of the standard error of predictions (Anderson and Raza 2010).

We offer a few cautionary notes on the use of AUC, but recognize the lack of alternatives for PO models (Hernandez et al. 2006, Lobo et al. 2008). For PO data, high AUC values indicate that the model can distinguish between presences and potentially unsampled locations (background), which is not necessarily a relevant distinction because the background sample contains both presences and absences. AUC penalizes for prediction beyond presence locations, which may be misleading when modeling a potential distribution, particularly if sampling effort is low. Since AUC is rank-based, comparisons among models are only valid when those models were built for the same landscape, background sample and species while using the same test data (Lobo et al. 2008, Elith et al. 2011). AUC increases when including more background locations that are trivial to distinguish from presence locations, but does not provide any additional ecological information, and can produce misleading measures of fit (Lobo et al. 2008, Anderson 2012). Thus AUC is most appropriate for species near range equilibrium, when sampling intensity is high, and background choice is based on biology.

Thresholding is problematic because choosing biologically meaningful thresholds may depend on prevalence or population density, which is typically unknown. Thus, arbitrary threshold values should not be used (e.g. interpreting logistic output >0.5 to mean more likely than not). Measures based on specificity (the proportion of absences correctly predicted) should be avoided because background points are not equivalent to absences (e.g. Kappa and the True Skill Statistic). Thresholding is unnecessary in many applications, and embracing the continuous and probabilistic nature of predictions avoids undue confidence in predictions. Often, thresholded predictions reflect researcher's assumptions about appropriate threshold values and not attributes of the species distribution.

IV. Conclusions

Differences in background sample selection, feature selection, sampling bias, and model output and evaluation fundamentally influence biological inference. This is precisely why Phillips and colleagues have gone to the trouble of allowing such flexible settings in MaxEnt. However, choosing MaxEnt settings in relation to the specific questions and data limitations should be the default approach rather than the current standard practice of simply adopting the default settings.

We suggest the following general protocol, in combination with our more specific suggestions above. First, we emphasize that modeling decisions should be taxon-specific and study goal-specific, and that our recommendations represent only a starting point. Second, we emphasize that one should always explore how different setting choices affect predictions and report these; if predictions show substantial differences across settings, it is critical to have a strong justification for the specific setting choice. Finally, the difficulty in evaluating PO models (section III.F) highlights the need for strong a priori justification of settings; while we may be unable to objectively evaluate a model we can ensure that it accurately reflects our assumptions and hypotheses.

Each of the decisions discussed in section III must be addressed for any modeling exercise. Sampling bias represents the greatest challenge for PO models. Sampling bias fundamentally disguises the biological pattern of interest, while other modeling decisions only affect the representation of that pattern. Conditional on accounting for sampling bias, we highlight some specific challenges for using MaxEnt for four common types of studies.

1) Projecting future species distributions. Projecting future species distributions, typically under climate change scenarios, usually involves extrapolating models to novel combinations of environmental variables (Elith et al. 2010, Webber et al. 2011). We believe such projections should be treated with extreme caution when derived from PO models. MaxEnt functional forms can either be 'clamped' at constant probabilities when projected into novel environments (e.g. Fig. 1) or they can simply be extended (in environmental space). We suspect that both options are unlikely to reflect biological reality in many, if not most cases. It is also critical to appreciate how the different assumptions made about background sampling and functional forms for response curves might influence extrapolated predictions. Insights into the sensitivity of extrapolated projections to different assumptions could be obtained by analyzing the predictions from an ensemble of models that consider the range of possible assumptions. We advise against using future projections as 'data' for subsequent analyses without recognizing these limitations.

2) Characterizing the niche and interpreting the influence of predictors on the distribution. When interest lies in the importance of predictors, it is critical that models have response curves that are sufficiently simple to be readily interpreted. The features retained by complex models are sensitive to which other predictors are included in the model

(Supplementary material Appendix 4, Table D1). Simple models allow one to inspect coefficients to infer importance and, given the relationship between MaxEnt and generalized linear models (Renner and Warton 2012), are more amenable to hypothesis testing to distill the primary environmental drivers of species' ranges.

3) Planning conservation measures. For conservation planning, it is typically important to know where the species actually occurs. This could lead to a tendency to produce models that predict occurrences well, but at the expense of complex response curves and potential over-fitting. We recommend against using MaxEnt to obtain the most accurate occurrence predictions without controlling for model complexity and consequent over-fitting. We also recommend against the common practice of thresholding predictions to identify predicted presences, due to the challenge of predicting probability of presence from the RORs produced by MaxEnt. Obtaining a threshold may be necessary for certain applications, in which case we advise reporting the sensitivity of the result to the chosen threshold and avoid at all costs drawing conclusions that rely on the adoption of a specific threshold. In spite of these limitations, PO data are sufficient for applications that require ranking locations by suitability. However, habitat suitability predictions can indicate where the species is most likely to occur but it cannot determine, e.g. whether the best habitat contains the species in 90% of samples, or only 10%. This is particularly problematic when trying to delimit a range boundary or the likelihood of finding an individual, and is compounded when trying to combine predictions for multiple species to obtain estimates of diversity (cf. Raes et al. 2009).

4) Understanding macroecological patterns. Studies of macroecological patterns typically involve running the same analyses on many species. However, time and resource constraints can make it impractical to perform species-specific tuning (Phillips et al. 2006). In such circumstances, one could argue for using default settings, although we advise against this. The large datasets used in macroecological studies are usually obtained from diverse data sources which may be likely to be contain sampling bias (e.g. museum data). Fortunately, the large data repositories used in macroecological studies naturally lend themselves to building TGS models for sampling bias. We strongly recommend investigating the sources of sampling bias within the different datasets in case it differs between species (Syfert et al. 2013). It is also likely to be impractical to closely inspect the response curves for individual species in macroecological studies. We therefore advise building simpler models with fewer feature classes and using stronger regularization to minimize the chances of overfitting.

The ease of changing MaxEnt's default settings allows modelers to better explore their data. For an overwhelming proportion of Earth's biodiversity, only PO data is available, so the best option is often to build PO models carefully while understanding their limitations. However, due to issues with sampling bias, PO data are primarily useful for exploratory analyses, which can help to inform structured survey strategies (e.g. PA or demographic) or help to formulate hypotheses. PO data, and consequently MaxEnt, may be best used for helping to ask better questions instead of answering them.

Acknowledgements – We thank Jane Elith, Kent Holsinger, Cynthia Jones, Andrew Latimer, Steven Phillips, Jorge soberon, Adam Wilson, and Niklaus Zimmerman for providing valuable suggestions for improving the manuscript and Tony Rebelo for providing the Protea Atlas data.

References

Aarts, G. et al. 2012. Comparative interpretation of count, presence–absence and point methods for species distribution models. – Methods Ecol. Evol. 3: 177–187.

Anderson, R. P. 2012. Harnessing the world's biodiversity data: promise and peril in ecological niche modeling of species distributions. – Ann. N. Y. Acad. Sci. 1260: 66–80.

Anderson, R. and Raza, A. 2010. The effect of the extent of the study region on GIS models of species geographic distributions and estimates of niche evolution: preliminary tests with montane rodents (genus Nephelomys) in Venezuela. – J. Biogeogr. 37: 1378–1393.

Anderson, R. P. and Gonzalez, I. 2011. Species-specific tuning increases robustness to sampling bias in models of species distributions: an implementation with MaxEnt. – Ecol. Model. 222: 2796–2811.

Araujo, M. and Peterson, A. 2012. Uses and misuses of bioclimatic envelope modelling. – Ecology in press.

Austin, M. 2002. Spatial prediction of species distribution: an interface between ecological theory and statistical modelling. – Ecol. Model. 157: 101–118.

Austin, M. 2007. Species distribution models and ecological theory: a critical assessment and some possible new approaches. – Ecol. Model. 200: 1–19.

Baasch, D. et al. 2010. An evaluation of three statistical methods used to model resource selection. – Ecol. Model. 221: 565–574.

Barve, N. et al. 2011. The crucial role of the accessible area in ecological niche modeling and species distribution modeling. – Ecol. Model. 222: 1810–1819.

Burnham, K. and Anderson, D. R. 2002. Model selection and multimodel inference: a practical information-theoretic approach, 2nd ed. – Springer.

Chakraborty, A. et al. 2011. Point pattern modelling for degraded presence-only data over large regions. – J. R. Stat. Soc. C 60: 757–776.

Cressie, N. A. C. 1993. Spatial statistics for spatial data. – Wiley-Interscience.

Dudik, M. and Phillips, S. 2009. Generative and discriminative learning with unknown labeling bias. – Adv. Neural Inform. Process. Syst. 21: 1–8.

Dudik, M. et al. 2004. Performance guarantees for regularized maximum entropy density estimation. – Learn. Theory Proc. 3120: 472–486.

Dudik, M. et al. 2005. Correcting sample selection bias in maximum entropy density estimation. – Adv. Neural Inform. Process. Syst. 17: 1–8.

Elith, J. et al. 2010. The art of modelling range-shifting species. – Methods Ecol. Evol. 1: 330–342.

Elith, J. et al. 2011. A statistical explanation of MaxEnt for ecologists. – Divers. Distrib. 17: 43–57.

Fithian, W. and Hastie, T. 2012. Finite-sample equivalence of several statistical models for presence-only data. – <http://arxiv.org/abs/1207.6950v1>.

Giovanelli, J. G. R. et al. 2010. Modeling a spatially restricted distribution in the Neotropics: how the size of calibration area affects the performance of five presence-only methods. – Ecol. Model. 221: 215–224.

Goodman, J. 2003. Exponential priors for maximum entropy models. – Technical report, Microsoft Research.

Graham, C. et al. 2004. New developments in museum-based informatics and applications in biodiversity analysis. – Trends Ecol. Evol. 19: 497–503.
Hastie, T. et al. 2009. The elements of statistical learning: data mining, inference, and prediction. – Springer.
He, F. 2010. Maximum entropy, logistic regression, and species abundance. – Oikos 119: 578–582.
Hernandez, P. A. et al. 2006. The effect of sample size and species characteristics on performance of different species distribution modeling methods. – Ecography 29: 773–785.
Hijmans, R. J. 2012. Cross-validation of species distribution models: removing spatial sorting bias and calibration with a null model. – Ecology 93: 679–688.
Hijmans, R. J. et al. 2005. Very high resolution interpolated climate surfaces for global land areas. – Int. J. Climatol. 25: 1965–1978.
Jaynes, E. 2003. Probability theory: the logic of science. – Cambridge Univ. Press.
Johnson, C. J. et al. 2006. Resource selection functions based on use-availability data: theoretical motivation and evaluation methods. – J. Wildl. Manage. 70: 347–357.
Keating, K. and Cherry, S. 2004. Use and interpretation of logistic regression in habitat-selection studies. – J. Wildl. Manage. 68: 774–789.
Kery, M. et al. 2010. Predicting species distributions from checklist data using site-occupancy models. – J. Biogeogr. 37: 1851–1862.
Latimer, A. et al. 2006. Building statistical models to analyze species distributions. – Ecol. Appl. 16: 33–50.
Lele, S. R. and Keim, J. L. 2006. Weighted distributions and estimation of resource selection probability functions. – Ecology 87: 3021–3028.
Linder, H. 2005. Evolution of diversity: the Cape flora. – Trends Plant Sci. 10: 536–541.
Liu, C. et al. 2005. Selecting thresholds of occurrence in the prediction of species distributions. – Ecography 28: 385–393.
Liu, C. et al. 2010. Measuring and comparing the accuracy of species distribution models with presence–absence data. – Ecography 34: 232–243.
Lobo, J. et al. 2008. AUC: a misleading measure of the performance of predictive distribution models. – Global Ecol. Biogeogr. 17: 145–151.
Manly, B. F. J. et al. 2002. Resource selection by animals: statistical analysis and design for field studies, 2nd ed. – Kluwer.
Newbold, T. et al. 2010. Testing the accuracy of species distribution models using species records from a new field survey. – Oikos 119: 1326–1334.
Peterson, A. T. et al. 2011. Ecological niches and geographic distributions. – Princeton Univ. Press.
Phillips, S. and Dudik, M. 2008. Modeling of species distributions with MaxEnt: new extensions and a comprehensive evaluation. – Ecography 31: 161.
Phillips, S. and Elith, J. 2010. POC plots: calibrating species distribution models with presence-only data. – Ecology 91: 2476–2484.
Phillips, S. et al. 2006. Maximum entropy modeling of species geographic distributions. – Ecol. Model. 190: 231–259.
Phillips, S. et al. 2009. Sample selection bias and presence-only distribution models: implications for background and pseudo-absence data. – Ecol. Appl. 19: 181–197.

Supplementary material (Appendix ECOG-07872 at <www.oikosoffice.lu.se/appendix>). Appendix 1–6.

Ponder, W. et al. 2001. Evaluation of museum collection data for use in biodiversity assessment. – Conserv. Biol. 15: 648–657.
Raes, N. et al. 2009. Botanical richness and endemicity patterns of Borneo derived from species distribution models. – Ecography 32: 180–192.
Rebelo, T. 2001. A field guide to the Proteas of southern Africa. – Fernwood Press.
Rebelo, T. 2002. The Protea Atlas Project – technical report. – <http://protea.worldonline.co.za/default.htm>.
Reddy, S. and Dávalos, L. 2003. Geographical sampling bias and its implications for conservation priorities in Africa. – J. Biogeogr. 30: 1719–1727.
Renner, I. W. and Warton, D. I. 2012. Equivalence of MAXENT and Poisson point process models for species distribution modeling in ecology. – Biometrics in press.
Royle, J. A. et al. 2012. Likelihood analysis of species occurrence probability from presence-only data for modelling species distributions. – Methods Ecol. Evol. in press.
Sastre, P. and Lobo, J. M. 2009. Taxonomist survey biases and the unveiling of biodiversity patterns. – Biol. Conserv. 142: 462–467.
Schulze, R. 1997. South African atlas of agrohyrdology and climatology. – Tech. Rep., Report TT82/96, Water Research Commission, Pretoria, South Africa
Syfert, M. et al. 2013. Accounting for sampling bias can dramatically improve the predictive accuracy of presence-only species distribution models. – PloS One in press.
Tibshirani, R. 1996. Regression shrinkage and selection via the lasso. – J. R. Stat. Soc. B 58: 267–288.
VanDerWal, J. et al. 2009. Selecting pseudo-absence data for presence-only distribution modeling: how far should you stray from what you know? – Ecol. Model. 220: 589–594.
Ward, G. et al. 2009. Presence-only data and the EM algorithm. – Biometrics 65: 554–563.
Warren, D. and Seifert, S. 2011. Ecological niche modeling in MaxEnt: the importance of model complexity and the performance of model selection criteria. – Ecol. Appl. 21: 335–342.
Warren, D. et al. 2010. ENMTools: a toolbox for comparative studies of environmental niche models. – Ecography 33: 607–611.
Warton, D. I. and Shepherd, L. C. 2010. Poisson point process models solve the "pseudo-absence problem" for presence-only data in ecology. – Ann. Appl. Stat. 4: 1383–1402.
Webber, B. L. et al. 2011. Modelling horses for novel climate courses: insights from projecting potential distributions of native and alien Australian acacias with correlative and mechanistic models. – Divers. Distrib. 17: 978–1000.
Wenger, S. J. and Olden, J. D. 2012. Assessing transferability of ecological models: an underappreciated aspect of statistical validation. – Methods Ecol. Evol. in press.
Wisz, M. S. and Guisan, A. 2009. Do pseudo-absence selection strategies influence species distribution models and their predictions? An information-theoretic approach based on simulated data. – BMC Ecol. 9: 8.
Yackulic, C. B. et al. 2012. Presence-only modelling using MAXENT: when can we trust the inferences? – Methods Ecol. Evol. in press.
Yates, C. J. et al. 2010. Assessing the impacts of climate change and land transformation on Banksiain the South West Australian Floristic Region. – Divers. Distrib. 16: 187–201.
