# Supplementary material for: Protected areas network is not adequate to protect a critically endangered East Africa Chelonian: Modelling distribution of pancake tortoise, Malacochersus tornieri under current and future climates
Source: PLoS One. 2021 Jan 20;16(1):e0238669. doi: 10.1371/journal.pone.0238669 (PMC7816999; doi:10.1371/journal.pone.0238669)
Supplement: Attachment — Submitted filename: Hannah et al 2020 Extinction risk mitigation.pdf [file pone.0238669.s005.pdf]

ECOGRAPHY

Research

30% land conservation and climate action reduces tropical extinction risk by more than 50%

Lee Hannah, Patrick R. Roehrdanz, Pablo A. Marquet, Brian J. Enquist, Guy Midgley, Wendy Foden, Jon C. Lovett, Richard T. Corlett, Derek Corcoran, Stuart H. M. Butchart, Brad Boyle, Xiao Feng, Brian Maitner, Javier Fajardo, Brian J. McGill, Cory Merow, Naia Morueta-Holme, Erica A. Newman, Daniel S. Park, Niels Raes and Jens-Christian Svenning

L. Hannah and P. R. Roehrdanz (https://orcid.org/0000-0003-4047-5011) ✉ (proehrdanz@conservation.org), The Moore Center for Science, Conservation International, 2011 Crystal Dr., Arlington, VA 22202, USA. – P. A. Marquet, D. Corcoran and J. Fajardo (https://orcid.org/0000-0002-0990-9718), Dept de Ecología, Facultad de Ciencias Biológicas, Pontificia Univ. Católica de Chile, Santiago, Chile. PAM, DC and JF also at: Instituto de Ecología y Biodiversidad (IEB), Santiago, Chile. – PAM and B. J. Enquist, The Santa Fe Institute, USA, Santa Fe, NM, USA. – BJE, B. Boyle, X. Feng, B. Maitner and E. A. Newman (https://orcid.org/0000-0001-6433-8594), Dept of Ecology and Evolutionary Biology, Univ. of Arizona, Tucson, AZ, USA. – G. Midgley, Dept of Botany and Zoology, Stellenbosch Univ., Stellenbosch, South Africa. – W. Foden, Cape Research Centre, South African National Parks, Cape Town, South Africa. – J. C. Lovett, School of Geography, The Univ. of Leeds, Leeds, UK, and: Royal Botanic Gardens, Kew, Richmond, Surrey, UK. – R. T. Corlett, Centre for Integrative Conservation, Xishuangbanna Tropical Botanical Garden, Chinese Academy of Sciences, Menglun, Yunnan, China. – S. H. M. Butchart, BirdLife International, David Attenborough Building, Pembroke Street, Cambridge, UK, and: Dept of Zoology, Univ. of Cambridge, Cambridge, UK. – B. J. McGill, School of Biology and Ecology, and Senator George J. Mitchell Center of Sustainability Solutions, Univ. of Maine, Orono, ME, USA. – C. Merow, Dept of Ecology and Evolutionary Biology, Univ. of Connecticut, CT, USA. – N. Morueta-Holme (https://orcid.org/0000-0002-0776-4092), Center for Macroecology, Evolution and Climate; GLOBE Institute; Univ. of Copenhagen, Copenhagen, Denmark. – D. S. Park (https://orcid.org/0000-0003-2783-530X), Dept of Organismic and Evolutionary Biology, Harvard Univ., MA, USA. – N. Raes (https://orcid.org/0000-0002-4329-4892), Naturalis Biodiversity Center, Leiden, the Netherlands. – J.-C. Svenning (https://orcid.org/0000-0002-3415-0862), Center for Biodiversity Dynamics in a Changing World (BIOCHANGE), Dept of Biology, Aarhus Univ., Aarhus, Denmark.

Ecography
43: 1–12, 2020
doi: 10.1111/ecog.05166

Subject Editor and
Editor-in-Chief: Miguel Araújo
Accepted 21 February 2020

Limiting climate change to less than 2°C is the focus of international policy under the climate convention (UNFCCC), and is essential to preventing extinctions, a focus of the Convention on Biological Diversity (CBD). The post-2020 biodiversity framework drafted by the CBD proposes conserving 30% of both land and oceans by 2030. However, the combined impact on extinction risk of species from limiting climate change and increasing the extent of protected and conserved areas has not been assessed. Here we create conservation spatial plans to minimize extinction risk in the tropics using data on 289 219 species and modeling two future greenhouse gas concentration pathways (RCP2.6 and 8.5) while varying the extent of terrestrial protected land and conserved areas from <17% to 50%. We find that limiting climate change to 2°C and conserving 30% of terrestrial area could more than halve aggregate extinction risk compared with uncontrolled climate change and no increase in conserved area.

Keywords: area-based conservation, biodiversity, climate change, conservation planning, extinction risk

NORDIC SOCIETY OIKOS

www.ecography.org

© 2020 The Authors. Ecography published by John Wiley & Sons Ltd on behalf of Nordic Society Oikos
This is an open access article under the terms of the Creative Commons Attribution License, which permits use, distribution and reproduction in any medium, provided the original work is properly cited.

Introduction

Preventing human-driven extinctions of the species sharing the planet with us is among the greatest environmental challenges of our time (Ehrlich and Mooney 1983, Rockström et al. 2009). The Convention on Biological Diversity (CBD) has been agreed by 195 nations to require '…maintenance and recovery of viable populations of species' for the conservation of biodiversity (Convention on Biological Diversity 1992). In the first draft of the post-2020 biodiversity framework developed by the CBD, extinction risk reduction is a goal for 2030 to achieve the longer-term 2050 vision of a world of 'living in harmony with nature' (CBD Secretariat 2020). Global extinction rate is a benchmark for planetary boundaries, defining both the sixth mass extinction event and a possible boundary for the Anthropocene geological epoch (Rockström et al. 2009, Dirzo et al. 2014, Ceballos et al. 2015).

Conservation of natural areas and sustainable use of nature are recognized as important instruments with which extinctions and other loss of biodiversity may be avoided (Convention on Biological Diversity 1992). A key target of the draft post-2020 biodiversity framework is to conserve of sites of importance for biodiversity through protected and conserved areas covering at least 30% of land and ocean areas (CBD Secretariat 2020). Both conservation and sustainable use are currently challenged by increasing large-scale monoculture and industrial developments, (Austin et al. 2017) making some form of conservation or land-use management essential to realizing these aims in most regions. Without such conservation, loss of species' habitats will result in increased extinction risk.

In addition to the local-to-global benefit of reducing extinctions and maintaining biodiversity, maintaining natural systems provides a wealth of benefits at all scales. A well-managed system of conserved areas can provide vital ecosystem services, such as water purification and retention, erosion control, the reduction of flooding, maintaining river base flows and opportunities for ecotourism as well as minimizing extinctions (Dudley and Stolton 2003, Mulongoy and Gidda 2008). For example, protected areas containing forests provide an important supply of drinking water to more than a third of the world's 100 largest cities (Dudley and Stolton 2003). The conservation and effective management of natural areas is currently recognized as an important nature-based solution for climate change mitigation (Griscom et al. 2017, Dudley et al. 2018, Marquet et al. 2019); these areas account for approximately 20% of the CO_2 sequestered by all terrestrial ecosystems (Melillo et al. 2016).

Climate change is now altering ecological conditions across the planet, both on land and in the oceans, and species are shifting their distributions in response to these new conditions (Bellard et al. 2012, Urban 2015, IPBES 2019). Species' distributions are determined by a combination of climatic and other niche tolerances (including edaphic), geographical barriers and competition, so shifting climatic conditions result in changes in the areas occupied by species, altering representation in conserved areas and changing or reducing ecosystem benefits (Foden et al. 2007, Lenoir et al. 2008, Pecl et al. 2017). Climate change is causing some species to shift their distributions out of existing conserved areas (Araújo et al. 2004, Heller and Zavaleta 2009, Johnston et al. 2013, Urban 2015), while also enabling some species to colonize new areas (Chen et al. 2011, Angelo and Daehler 2013). Species' climate niches are unique, meaning that species move individualistically in response to climate change. Species will be moving at different rates and in different directions, resulting in altered combinations of species in any one location over time.

The interaction between climate change and complex landscapes makes knowing where to conserve species more complicated. Conservation plans that minimize area requirements based on species' current locations and habitat requirements will no longer be effective as those locations and habitats change. Establishing protected and conserved areas in the right places to facilitate species' distributional shifts through time can help avoid extinctions due to climate change (Williams et al. 2005, Hannah et al. 2007, Phillips et al. 2008, Alagador et al. 2016, Bagchi et al. 2018). Until now it is unknown how much extinction risk reduction is possible under different extents of conserved area considering the effects of both land use change and climate change (though see Baillie and Zhang 2018).

To address these questions, we created conservation spatial plans for 289 219 tropical plant and vertebrate species that minimized both present and modeled future extinction risk under two greenhouse gas concentration pathways – RCP2.6 and 8.5 – across varying area of terrestrial conservation, from current levels up to 50% conserved including 30% conserved as suggested in the draft post-2020 framework (CBD 2020). We used widely available vertebrate distribution and occurrence data as well as the most up-to-date compilation of terrestrial plant data (Enquist et al. 2019). Extinction risk results are expressed as the mean, maximum and minimum of our 10-climate model ensemble. Extinction risk can be assessed in multiple ways. The IUCN Red List classifies species into different categories of relative extinction risk, while multiple modeling approaches exist for estimating probability of extinction including the species area relationship (SAR), The Zonation conservation planning software (Moilanen et al. 2014) incorporates a theoretical SAR to produce estimates of extinction risk for every species. We sum these values across species to calculate an aggregate extinction risk (AER, ranging from 0 if no species are at risk to 1 if all species have gone extinct) and summarize this for three major tropical biogeographic regions – the Neotropics, Afrotropics, and Southeast Asian tropics for both climate scenarios and conservation from present to 50% of terrestrial area. This allows us to assess, for the first time, which natural areas are required to minimize extinction risk of tropical species in the face of climate change and how much extinction risk we may avoid in conserving additional area.

Material and methods

Overview

We modelled present and future geographic ranges of 94 337 plant species and 9722 vertebrate species across the Neotropics, Afrotropics and Southeast Asian tropics. Our methods are described in detail below, including the following methods: 1) climate models and RCPs – we used 10 GCM selected to be consistent across the three regions and two RCP (2.6 and 8.5) to provide low and high brackets of future climate; 2) spatial planning algorithm – we used Zonation conservation planning software with settings customized to accommodate range shifts under climate change; 3) extinction risk calculation – we used an AER index equal to the sum of individual species' extinction risks, using a SAR; 4) species distribution models – we used Maxent (Phillips et al. 2006) to fit species distribution models at 30 arc-second (~1 km) resolution then project distributions into baseline climate all future climate scenarios at 2.5 arc-minutes (~5 km) for analysis; 5) environmental predictor variables – were taken from standard WorldClim variables plus a custom aridity index; 6) soils data – global soilgrids variables; 7) land use and land cover data – from global consensus landcover dataset; 8) plant data – we used botanical occurrence data assembled and standardized within the BIEN database; 9) vertebrate data – we used publicly available occurrence records verified with IUCN expert range polygons. Details are given below for each of these methods.

Climate models and RCPs

We used 10 GCMs and two climate scenarios for the 2060–2080 timeframe. The GCMs used to drive our biological models include Access 1.0, BCC-CSM1.1, CNRM-cm5, GFDL-cm3, MOHC-HADGEM2-es, NCAR-CCSM4, LASG-FGOALS-g2, NCC-NORESM-m, MIROC-ESM and MPI-ESM-lr. We used a low forcing scenario, RCP2.6, to approximate climate change consistent with meeting the Paris Agreement target of 2.0° global mean temperature change, and RCP8.5 to approximate business-as-usual, no action on climate change.

Spatial planning algorithm

We used the Zonation Conservation Planning Software ('Zonation'), a tool that allows for simultaneous prioritization of many thousands of conservation features (Moilanen et al. 2005, Moilanen 2007). We applied Zonation at a horizontal spatial resolution of 2.5 arc-minutes (~5 km). Zonation can be used for conservation prioritization under climate change. This configuration of Zonation allows for simultaneous prioritization of a species current range, its modelled future range, and the connectivity between the two limited by a species' capacity to disperse. The protocol for running Zonation under climate change projections is described in Kujala et al. (2013). Species current ranges were linked to projected future ranges through an interaction layer (Kujala et al. 2013). This layer is transformed by a dispersal kernel with a parameter to limit the interaction to the species total capacity to disperse over the period of analysis. Total dispersal capacity was assumed to be 100 km for vertebrates (roughly 1 km year^{-1}) and 10 km for vascular plants (roughly 0.1 km year^{-1}) following previously published studies (Warren et al. 2013). Species with too few occurrence records to produce a model were included as point locations (Zonation term = 'species of special interest' or SSI) where the range was defined as each grid cell containing ≥1 occurrence record. Equal weighting was used for all conservation features.

Extinction risk calculation

Zonation/climate seeks to maximize biodiversity representation – both present and future – and thereby minimize extinction risk, at each area increment, based on a power-law species area relationship (SAR) parameter that can be set for each conservation feature. Here we used the default SAR parameter (z = 0.25) for all areas in order to transform of the proportion of species ranges conserved at each step of the solution to the per species extinction risk where:

$$\text{Extinction risk} = 1 - \left(\text{Proportion conserved} \wedge 0.25\right)$$

To summarize over all species at different time steps, we calculated aggregate extinction risk (AER) at each step as the sum of individual species' remaining extinction risk across all species normalized by the total number of species.

Species distribution models

Species distribution models were produced with Maxent (Phillips et al. 2006) for species with >10 unique occurrence records (i.e. unique 1 km grid cells in the modelling domain) (van Proosdij et al. 2016). Maxent settings followed the recommendations of Merow et al. (2013, 2014) to produce relatively less complex models (e.g. limiting features to linear, quadratic, and product functions) to minimize overfitting. Modelling domains were limited to a spatial buffer of within 500 km of any valid occurrence record. This likewise limited the projected ranges to within 500 km of any verified observation. Background sampling was a random sample of 10 000 points within the buffered modelling domain. Five model replicates were used in fitting the model and an average of the five replicates was used for final species model. Parameters from the final model were used to project species suitability for both baseline and future climate scenarios. Default values for regularization coefficients were used and 30% of occurrence records were randomly reserved to assess model performance.

Environmental predictor variables

We chose the following bioclimatic variables from downscaled 20-year normals (Hijmans et al. 2005) baseline (1960–1990) and future (2060–2080) climates based on pan-tropical correlation analysis: mean annual temperature (BIO1), mean diurnal temperature range (BIO2), seasonality of temperature (BIO4), minimum temperature of the coldest month (BIO6), mean annual precipitation (BIO12), seasonality of precipitation (BIO15). We also used an accumulated aridity index that is the sum of the monthly aridity (annual precipitation – PET) for the maximum run of consecutive months where (PET > precipitation). Accumulated aridity index is derived from global monthly extra-terrestrial solar radiation data from Trabucco and Zomer (2019) and monthly maximum temperature, minimum temperature and precipitation from Worldclim ver. 1.4 (Hijmans et al. 2005).

Soils data

Soils variables used in species distribution models were depth to bedrock, pH, clay proportion, silt proportion and bulk density. All soil-related variables were obtained from Soilgrids ver. 1.0 (accessed February 2018) (Hengl et al. 2017). Variables with multiple strata available are the mean of the top 1 m (strata 1–4). Soils-variables were included as it has been shown that climate change analyses that do not incorporate soils variability can misrepresent edaphic specialists (Corlett and Tomlinson 2020).

Land cover and land use data

Areas of existing built up land or intensive agriculture were removed from the analysis and therefore those cells are not part of the prioritization solution. Built up and agricultural areas were defined as >50% of pixel coverage for 'urban' and 'agriculture' classes from the 1 km resolution global consensus land cover dataset produced by Tuanmu and Jetz (2014) that were aggregated to match the 2.5 arc-minute resolution of analysis. Existing protected areas (IUCN and WCMC 2019) were solved first using a hierarchical mask so that the conservation priorities take the effects existing protection into account. The analysis domain was limited to the Afrotropics, Neotropics and Indo-Malayan biogeographic realms with the Indonesian plus Papua New Guinea portion of the Australasian realm also included in the Asia domain (Dinerstein et al. 2017).

Plant data

Vascular plant data was extracted from the BIEN ver. 4.1 database using the RBIEN package (Maitner et al. 2018). Occurrence records with geographic information were obtained for 275 372 species of which we modelled 94 337. The BIEN data mainly comprise herbarium collections, ecological plots and surveys (DeWalt et al. 1999, Wiser et al. 2001, Enquist et al. 2012, 2016, Fegraus 2012, Peet et al. 2012, Forest Inventory and Analysis National Program 2013, Anderson-Teixeira et al. 2015), and trait observations. For details of specimen data sources see Maitner et al. (2018). A full listing of the herbaria data used are given in the Acknowledgements section. The observations in the BIEN database are the product of contributions by 1076 different data contributors, including numerous individual herbaria, data indexers of herbarium or plot data. Of the herbaria, 550+ are listed in Index Herbariorum. Additionally, BIEN 4.1 includes data from RAINBIO, TEAM, The Royal Botanical Garden of Sydney, Australia, and NeoTropTree. Plot data within BIEN are from the CVS, NVS, SALVIAS, VEGBANK, CTFS, FIA, MADIDI, and TEAM data networks and datasets (<http://bien.nceas.ucsb.edu/bien/data-contributors/all/>).

Vertebrate data

Point occurrences for tropical vertebrate species were compiled from GBIF (GBIF download 2019; accessed through R package 'rgbif') and restricted range bird species records (Birdlife International 2018) for 13 847 tropical species, 9722 of which had sufficient occurrence records (>10) for modelling (van Proosdij et al. 2016). Occurrences were filtered to include only those with specific georeferencing coordinates, observations more recent than 1950, and human observations only (no fossil records or museum specimens). Additionally, spatial outliers (more than 500 km from IUCN range polygon or >98th percentile of latitude + longitude) were removed prior to modelling. IUCN and Birdlife International range map polygons for all available terrestrial mammals, birds, reptiles were used to generate the species list and as a means of occurrence record validation (Birdlife International 2018, IUCN and UNEP WCMC 2018).

Results

Our results show that a large reduction in extinction risk can be achieved by moving to a world in which 30% or 50% of terrestrial area is conserved, corresponding to the proposed action targets of the first draft post-2020 CBD framework (CBD 2020). Figure 1 illustrates the modeled reduction in aggregate extinction risk with increasing area conserved for the Neotropics, Afrotropics and SE Asian tropics respectively. This reduction in extinction risk results from both reduced loss of habitat due to land use change and better representation of changing habitats for species moving in response to climate change. Outside of fully natural landscapes, achieving these minimum extinction results requires matching land uses to the habitat needs of species at risk of extinction in a particular area. For instance, shade coffee may provide habitat for many bird species, but not for understory tree species. Actual extinction risk may be somewhat higher because multiple-use or sustainable use landscapes may not provide suitable habitats for all species.

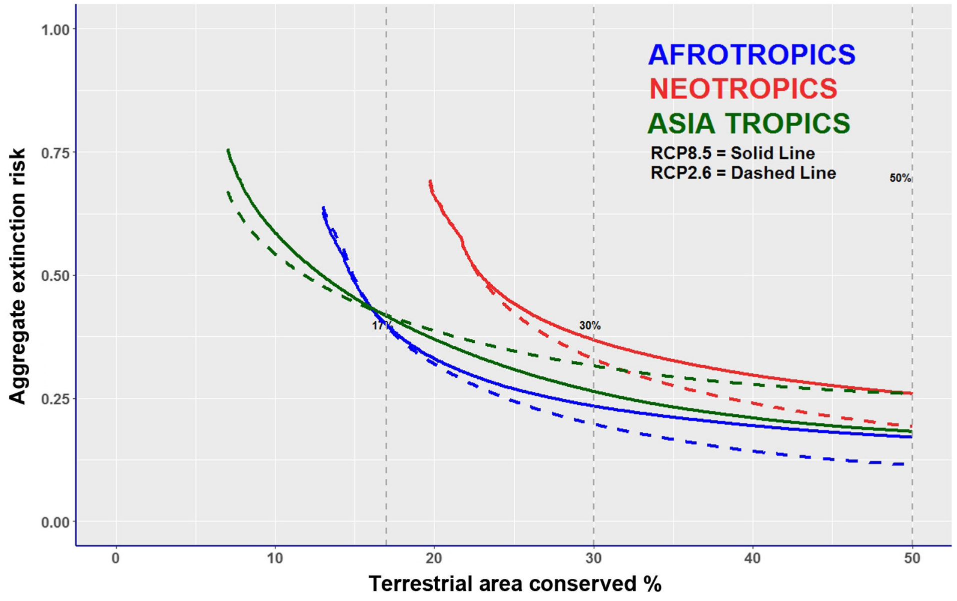

Figure 1. Modeled extinction risk reduction with increasing land conservation. Ensemble mean Aggregate extinction risk (AER) versus % terrestrial area conserved under RCP 2.6 (dashed lines) and RCP 8.5 (solid lines) for the Afrotropics (blue), Neotropics (red) and Asia Tropics (green). Vertical lines show the aggregate extinction risk curve intersection with 17%, 30% and 50% terrestrial area conserved. AER is the mean of individual species extinction risk at each increment of conserved natural land and is scaled from 0 to 1 (zero probability of extinction to likely extinct).

The spatial distribution of natural areas needed to achieve these reductions in extinction risk is illustrated for each of the three major tropical regions in our study in Fig. 2A–C. Many areas highlighted are recognized as global biodiversity hotspots (Myers et al. 2000), indicating the continued importance of conserving these regions of high endemism. Many also correspond to areas recently identified as plant rarity hotspots (Enquist et al. 2019). Mountain ranges and other areas of high topographic diversity feature prominently, likely due to existing high biodiversity, high endemism, and comparatively high climate and microclimate diversity (Rahbek et al. 2019a, b) coupled with the comparatively low velocity of climate change in these areas (Loarie et al. 2009). However, as the core area algorithm used (Moilanen 2007) prioritizes representation of all species (i.e. not just species associated with diverse ecosystems),and will try to link niche-tracking to areas already conserved to the greatest degree possible, spatial priorities capture many distinct ecosystems and climate types including lowland ecoregions.

The areas our prioritization highlighted as important for species on the move include many areas that are also high conservation priority under current climate. This is because conserving a species from present to future begins with conservation of the species' current range, since this serves as the starting point for any future dispersal. Restricted-range species are concentrated in mountainous areas (Rahbek et al. 2019a, b), where species' movements in response to climate change will generally be upslope with warming temperatures (Peters and Darling 1985, Halpin 1997) or into nearby areas with suitable microclimates in complex terrain (Hannah et al. 2014, Rahbek et al. 2019b). Low velocity of climate change in mountains (Loarie et al. 2009) and decreasing area with elevation mean that species' upslope movements will occur over shorter distances in mountains (Serra-Diaz et al. 2014), further concentrating restricted-range species near current montane centers of endemism (Enquist et al. 2019).

We observe a steep drop-off in modeled AER with increasing conservation of (primarily montane) natural area (Table 1). If conservation is limited to existing protected areas, AER is projected to be high under both climate change scenarios – ranging from 60 to 77% under both RCP 8.5 and 2.6. Moving to 30% land conservation combined with lowering climate change (RCP2.6) results in reduction of modeled AER by 52–68% (ensemble mean) across all regions when compared with existing levels of conservation and higher (RCP8.5) climate change. Unsurprisingly, the greatest extinction risk reduction could be achieved by conserving 50% of terrestrial area with a low climate change scenario; this combined action reduces modeled extinction risk by 72–82% in the ensemble mean (Table 1). Conserving 30% or 50% of land but with high climate change (RCP8.5), reduces modeled extinction risk between 46–66% and 62–76% respectively across the three regions under business-as-usual climate change (RCP 8.5) in the ensemble mean. Reducing climate change from business as usual (RCP 8.5) to the RCP 2.6 scenario which is more consistent with limiting mean global temperature change to 2.0° could reduce extinction risk 5–6% more under 30% land conservation. In contrast, with no increase in existing conservation area (<17% area), the same climate policy is projected to result in roughly −2 to 12% additional decrease in ensemble mean extinction risk.

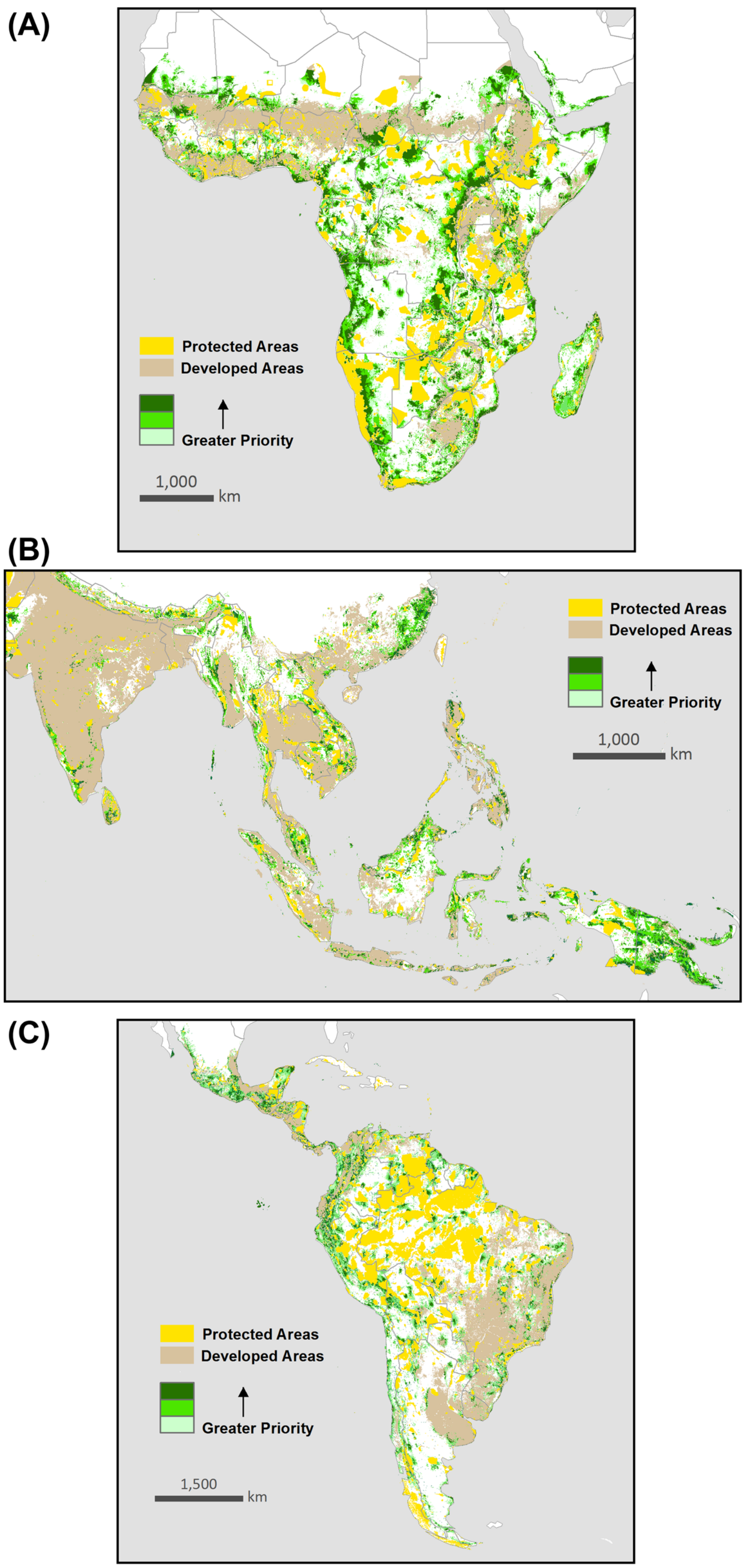

Figure 2. (A–C) Spatial prioritizations for land conservation to minimize extinction risk. Zonation spatial solutions to minimize extinction risk in the Afrotropics (A), SE Asia tropics (B) and Neotropics (C). AER values in Fig. 1 are derived from these spatial solutions. Areas of darkest green are highest priority areas to minimize extinction risk at 17% land conservation. Successively lighter shades of green represent areas that minimize extinction risk at 30% and 50% land conservation. Existing conserved areas registered in the World Database of Protected Areas are shown in yellow. Priorities are selected to maximize representation of all species in both baseline (1960–1990) and future ranges (RCP8.5 2060–2080).

Table 1. Aggregate extinction risk (AER) under varying land conservation areas and climate change. Ensemble mean, maximum and minimum AER for high (RCP 8.5) and low (RCP 2.6) climate scenarios and three levels of land conservation (existing protected areas, 30% land conservation and 50% land conservation) for Asia, Africa and the Neotropics. Values are summed extinction risk across all species, scaled 0 (zero extinction risk for all species) to 1 (100% extinction risk for all species). The 'change' columns represent the percent difference in AER relative to the current protected areas under RCP 8.5. The extinction risk values in Table 1 are presented in continuous form in Fig. 1.

	Ensemble mean	Mean ensemble change	Ensemble max	Max ensemble change	Ensemble min	Min ensemble change
Neotropics						
RCP8.5						
Existing protected area*	0.686	–	0.765	–	0.631	–
30% land conservation#	0.370	−46.1	0.468	−38.8	0.332	−47.4
50% land conservation	0.259	−62.2	0.368	−51.9	0.219	−65.3
RCP2.6						
Existing protected area	0.693	−2.6	0.735	−3.4	0.675	−0.8
30% land conservation	0.332	−51.6	0.400	−58.2	0.301	−62.3
50% land conservation	0.192	−72.0	0.269	−74.9	0.159	−74.8
Afrotropics						
RCP8.5						
Existing protected area	0.628	–	0.657	–	0.601	–
30% land conservation	0.234	−62.7	0.267	−60.2	0.195	−67.6
50% land conservation	0.171	−72.8	0.217	−71.8	0.140	−76.7
RCP2.6						
Existing protected area	0.639	1.8	0.653	−0.6	0.629	4.7
30% land conservation	0.197	−68.6	0.220	−66.5	0.178	−70.4
50% land conservation	0.114	−81.8	0.140	−78.7	0.097	−83.9
Asia Tropics						
RCP8.5						
Existing protected area	0.756	–	0.771	–	0.745	–
30% land conservation	0.261	−65.5	0.300	−61.1	0.227	−69.5
50% land conservation	0.182	−75.9	0.223	−71.1	0.152	−79.6
RCP2.6						
Existing protected area	0.671	−11.2	0.762	−1.2	0.614	−17.6
30% land conservation	0.314	−58.5	0.360	−69.2	0.231	−69.0
50% land conservation	0.259	−65.7	0.313	−80.5	0.143	−80.8

* IUCN categories I–VI.
# IUCN categories I–VI + OECM.

Discussion

Our analysis suggests that 30% land conservation combined with climate change action could reduce extinction risk by two thirds or more across multiple conservation/climate combinations in all three tropical regions. Models show that with higher climate change (RCP8.5), 30% land conservation could reduce AER by at least half in the ensemble mean, except in the Neotropics. The lower levels of modeled reduction in the Neotropics are likely due to the high number of rare species in the Andes, distributed over a relatively large area that can't be completely captured with 30% land conservation. 30% land conservation could therefore pay major benefits to biodiversity conservation and is a strong target for CBD post-2020 consideration.

The analysis also suggests that 50% land conservation could lead to an even larger decrease in extinction risk, resulting in over 80% reduction in all regions when coupled with lower climate change (RCP2.6). Roughly half of the world's ecoregions have already lost 50% or more of their original natural habitat (Dinerstein et al. 2017) so this longer-term vision of 50% land conservation would require restoration in many areas. Careful targeting of this restoration can have important long-term benefits for biodiversity. Geographic targeting at coarse scales can be accomplished by selecting priority areas indicated in Fig. 2A–C. Finer-scale priorities can be further elaborated by conducting local systematic spatial planning for climate change using methods similar to this study.

Natural areas can be conserved through a wide range of mechanisms, from government-designated protected areas to multiple-use land management and other effective area-based conservation measures such as some community conservancies (Dudley et al. 2018, Dinerstein et al. 2019). Which approach is the most appropriate will depend on the local environmental and social context (Brown et al. 2003). What is clear is that large-scale industrial land uses, particularly monoculture and plantations, are becoming increasingly prevalent, so achieving the results reported here will require active policy or land use management intervention to maintain natural areas in high priority locations of highest value to species' present and future ranges.

The modeled reductions in extinction risk we report depend on specific spatial configurations in our conservation solutions that in turn depend on GCM and species model variants. The uncertainty associated with our GCM variants is relatively low. Across 10 general circulation models (GCM), the average ratio of ensemble mean to maximum is 0.85 and of ensemble mean to minimum is 1.17 for the 30% conserved area target. Species modelling uncertainty is higher than the uncertainty associated with climate models (Araújo and Guisan 2006). Modeling a large number of species over a broad spatial domain at relatively fine resolution requires substantial compute resources, which is then multiplied when several GCMs and climate pathways are considered. It is an acknowledged limitation of this study that we report the results of only one species distribution model method, as it has been found that choice of modeling method is often the largest source of variation in species range projections (Diniz-Filho et al. 2009, Garcia et al. 2012). Exploring solution space with multiple species models is important and is a priority for our research group and we recommend that the results presented here be vetted against alternative species modeling techniques. Use of ensemble species modeling methods and the uncertainties therein is a key recommendation of recently agreed upon standards for biodiversity assessments (Araújo et al. 2019) and is therefore an opportunity to refine the results presented here. Indeed, the approach described in Kujala et al. (2013) which was followed this study offers a template to incorporate species model uncertainty as well as climate model uncertainty in the prioritization.

Despite this limitation, there are multiple reasons for us to believe that these results, using only one species distribution model method (Maxent) are likely robust at the scale of our analysis. First, our conservation planning algorithm, Zonation, seeks solutions that minimize extinction risk, which means minimizing range loss in rare species. Rare species are concentrated in tropical and Mediterranean mountains (Enquist et al. 2019, Rahbek et al. 2019a, b). These areas will emerge as high priority under current and future climate, regardless of species model. Second, we include species that have too few records to be modeled. These species are included in the conservation prioritizations with their current occurrences set equal to their future occurrences. Since most species are rare (Enquist et al. 2019), nearly one-third of the species driving our priorities are these rare species with too few occurrences to model. These species and the spatial priorities to conserve them are independent of species models. Third, species will be predominantly moving upslope in tropical mountains, so while exact locations of conservation importance for individual species may be dependent on species models, general tropical montane locations, becoming even more concentrated as climate changes, dominate our general global results. Since rare plant species are common and >90% of our modelled species are plants, including non-modelled species reduces the biases associated with exclusive reliance on the more widespread species with enough records to model. Finally, Rare species are concentrated where past climate change could be tracked with limited movement (Sandel et al. 2011), likely reflecting frequent limited dispersal ability in these species (Foden et al. 2013) so at the spatial resolution of our study, their future ranges are likely to be within the same selection unit as their present range, reducing or eliminating the uncertainty that arises from lack of ability to simulate their future range.

The AER and spatial results reported here show that on coarse scales and across broad domains, we know where to conserve to be most effective in meeting international conservation goals, even as climate changes. Conserving the areas highlighted in our results can pay large dividends in carbon sequestration to reduce climate change, as well as providing other ecosystem services while reducing extinction risk and maintaining biodiversity. However, the converse is not necessarily true – maximizing carbon sequestration may not automatically conserve these critical biodiversity and climate priorities (Di Marco et al. 2015). Carbon sequestration, both above-ground and below-ground, may be higher in lowland ecosystems. A strategy to maximize per unit area carbon sequestration by pushing agriculture and other development into more marginal uplands will require clearing more habitat to meet production needs, in the very habitats that are most important to conserve (Di Marco et al. 2015, Rahbek et al. 2019a, b). In some cases, a strategy of conserving more upland area (to sequester the same amount of carbon) could meet the same carbon goal, provide substantial ecosystem services such as watershed protection, while having much greater biodiversity benefit and potentially, as tradeoff,

allowing some lowland areas with high agricultural potential to be developed.

Achieving these multiple biodiversity and ecosystem service benefits in the real world requires continuous, iterative planning. No set of priorities can be completely or instantaneously realized in a world of multiple competing development interests that play out over time. Rather, in the real world, conservation and development planning moves incrementally and often imperfectly. So while the spatial priorities presented here can greatly reduce extinction risk, real world planning to reduce extinction risk will need to accept compromises and reorder priorities as choices (many times less than optimal) are made. More important than a perfect set of priorities is an ongoing planning process that considers climate change effects on biodiversity and ecosystem services. Such a process can ensure that at every decision point about which areas are to remain natural or to be developed, the highest priority areas for biodiversity under climate change are identified. While the highest priority area may not always be conserved, systematic bias towards the highest priority remaining sites will progressively drive solutions towards reduction of extinction risk. For this reason, it is important to establish systematic conservation planning for biodiversity and climate change as a key process within government offices in charge of conservation and development planning, as they should continuously update priorities for conservation as solutions are implemented, as well as when knowledge of changes in species distributions and other effects of climate change increases.

Ongoing systematic conservation planning allows for assimilation of new data and improved climate models. For the conservation of biodiversity, uncertainties need not stand in the way of action. By building systematic planning processes that serve communities and nations for decades, progressive reduction in uncertainty will result in continually improved conservation and development outcomes. The perfect should not be the enemy of the good; our land use plans will never be perfect, but they can be good, they can incorporate consideration of conservation and species movements due to climate change and they can continually improve. Doing so can help ensure that all of the species that share this planet with us will continue to thrive, and provide benefits to people, even as climate changes.

Data availability statement

Data are available from the Dryad Digital Repository: <http://dx.doi.org/10.5061/dryad.xxxxx> (Hannah et al. 2019).

Acknowledgements – This work is supported by the Global Environment Facility grant GEF-5810 ‘Spatial Planning for Area Conservation in Response to Climate Change (SPARC)’. Additionally, this work was conducted as a part of the Botanical Information and Ecology Network (BIEN) Working Group, 2008–2012. PIs BJE, RC, BB, SD and RKP were supported by the National Center for Ecological Analysis and Synthesis, a center funded by NSF (grant no. EF-0553768), the Univ. of California, Santa Barbara, and the State of California. Additional support was provided for JCD, the NCEAS Research Associate in the Group. The BIEN Working Group was also supported by the iPlant Collaborative (NSF no. DBI-0735191). We thank all the data contributors, numerous herbaria who have contributed their data to various data compiling organizations for the invaluable data and support provided to BIEN. We especially thank the New York Botanical Garden, Missouri Botanical Garden, Biodiversity Center, Leiden, the Netherlands, the UNC Herbarium as well as GBIF, REMIB, and SpeciesLink. The staff at iPlant and the Texas Advanced Computing Center at the University of Texas provided critical computational assistance. We thank the more than 50 scientists have participated in our various BIEN working group and sub-group meetings since 2008 including B. Blonder, K. Engemann, E. Fegraus, J. Cavender-Bares, B. Dobrin, K. Gendler, R. Jorgensen, G. Lopez-Gonzalez, L. Zhenyuan, S. McKay, O. Phillips, J. Pickering, N. Swenson, C. Vriesendorp, K. Woods, who participated in a working group meeting and D. Ackerly, E. Garnier, R. Guralnick, W. Jetz, J. Macklin, N. Matasci, S. Ramteke and A. Zanne participated in sub-group meetings. We thank John J. Wiens and Michael Sanderson for prior comments and encouragement. We also acknowledge the critical support of iPlant from R. Jorgensen, S. Goff, N. Matasci and R. Walls. Further, the long-term vision, encouragement, and support of F. Davis, S. Hampton, M. Jones, and the ever-helpful staff at NCEAS were critical for the completion of this first stage of the BIEN working group. NMH was supported by the European Union’s Horizon 2020 research and innovation program under the Marie Sklodowska-Curie grant agreement No. 746334 and acknowledges the Danish National Research Foundation for support to the Center for Macroecology, Evolution and Climate (grant no. DNRF96). CV was supported by a Marie Curie International Outgoing Fellowship within the 7th European Community Framework Program (DiversiTraits project, no. 221060) by the European Research Council (ERC) Starting Grant Project (Grant ERC-StG-2014-639706-CONSTRAINTS). JCS and BJE acknowledge support from the Center for Informatics Research on Complexity in Ecology (CIRCE), funded by the Aarhus University Research Foundation under the AU Ideas program. XF and EAN were supported by the University of Arizona Bridging Biodiversity and Conservation Science program. C.M. acknowledges funding from NSF Grant DBI-1913673. JCS considers this work a contribution to his VILLUM Investigator project ‘Biodiversity Dynamics in a Changing World’ funded by VILLUM FONDEN (grant 16549). We acknowledge the herbaria that contributed data to this work: HA, FCO, DUKE, MFU, UNEX, VDB, ASDM, AMD, BPI, BRI, BRM, CLF, CNPO, L, LPB, AD, A, TAES, FEN, FHO, ANSM, ASU, B, BCMEX, RAS, RB, TRH, AAH, ACOR, UI, AK, CAS, ALCB, AKPM, EA, AAU, ALTA, ALU, AMES, AMNH, AMO, CHAPA, GH, ANGU, ANSP, ARAN, AS, CICY, BAI, CIMI, AUT, BA, BAA, BAB, CMMEX, BACP, BAF, BAJ, BAL, COCA, CODAGEM, BARC, BAS, BBS, BC, BCN, BCRU, BEREA, BG, BH, BIO, BISH, SEV, BLA, BM, BOCH, MJG, BOL, CVRD, BOLV, BONN, DAV, BOUM, BR, DES, BREM, BRLU, BSB, BUT, C, DS, CALI, CAN, CANB, CAY, EBUM, CBM, CEN, CEPEC, CESJ, CHR, ENCB, CIIDIR, CINC, CLEMS, F, COA, COAH, FCME, COFC, CP, COL, COLO, CONC, CORD, CPAP, CPUN, CR, CRAI, FURB, CU, G, CRP, CS, CSU, CTES, CTESN, CUZ, DAO, HB, DBN, DLF, DNA, DR, DUSS, E, HUA, EAC, EIF, EIU, GES, GI, GLM, GMNHJ, K, GOET, GUA, EMMA, HUAZ, ERA, ESA, FAA, FAU, FB,

UVIC, FI, GZU, H, FLAS, FLOR, HCIB, FR, FTG, FUEL, GB, HNT, GDA, HPL, GENT, HUAA, HUJ, CGE, HAL, HAM, IAC, HAMAB, HAO, HAS, IB, HASU, HBG, IBUG, HBR, HEID, IEB, HIP, IBGE, ICEL, ICN, ILL, SF, HO, HRCB, HRP, HSS, HU, HUAL, HUEFS, HUEM, HUFU, HUSA, HUT, IAA, HXBH, HYO, IAN, ILLS, HAC, IPRN, IMSSM, FCQ, ABH, INEGI, INIF, BAFC, BBB, INPA, IPA, NAS, INB, INM, MW, EAN, IZTA, ISKW, ISC, ISL, GAT, JEPS, IBSC, UCSB, ISTC, ISU, IZAC, JACA, JBAG, JE, SD, JUA, JYV, KIEL, ECON, KSC, TOYA, MPN, USF, TALL, RELC, CATA, AQP, KMN, KMNH, KOELN, KOR, FRU, KPM, KSTC, LAGU, TRTE, KSU, UESC, GRA, IBK, KTU, ACAD, MISSA, KU, PSU, KYO, LA, LOMA, LW, SUU, UNITEC, TASH, NAC, UBC, IEA, GMDRC, LD, M, LE, LEB, LIL, LINN, AV, HUCP, QFA, LISE, MBML, NM, MT, FAUC, MACF, CATIE, LTB, LISI, LISU, MEXU, LL, LOJA, LP, LPAG, MGC, LPD, LPS, IRVC, MICH, JOTR, LSU, LBG, WOLL, LTR, MNHN, CDBI, LYJB, MOL, DBG, AWH, NH, HSC, LMS, MELU, NZFRI, MA, UU, MU, CSUSB, MAF, MAK, MB, KUN, MARY, MASS, MBK, MBM, UCSC, UCS, JBGP, DSM, OBI, BESA, LSUM, FULD, MCNS, ICESI, MEL, MEN, TUB, MERL, CGMS, MFA, FSU, MG, HIB, MIL, DPU, TRT, BABY, ETH, YAMA, SCFS, SACT, ER, JCT, JROH, SBBG, SAV, PDD, MIN, SJSU, MMMN, PAMP, MNHM, OS, SDSU, BOTU, OXF, P, MOR, POM, MPU, MPUC, MSB, MSC, CANU, SFV, RSA, CNS, WIN, MSUN, CIB, MUR, MTMG, VIT, MUB, MVFA, SLPM, MVFQ, PGM, MVJB, MVM, MY, PASA, N, UCMM, HGM, TAM, BOON, UFS, MARS, CMM, NA, NU, UADY, UAMIZ, UC, NE, NHM, NHMC, NHT, UFMA, NLH, UFRJ, UFRN, ULS, UMO, UNL, UNM, US, NMB, NMNL, USP, NMR, NMSU, WIS, NSPM, XAL, NSW, NT, ZMT, BRIT, MO, NCU, NY, TEX, U, UNCC, NUM, O, CHSC, LINC, CHAS, ODU, CDA, OSA, OSC, OSH, OULU, OWU, PACA, PAR, UPS, PE, PEL, SGO, PEUFR, PFC, PH, PKDC, SI, PLAT, PMA, PORT, PR, QM, PRC, TRA, PRE, PY, QCA, TROM, QCNE, QRS, UH, QUE, R, SAM, RBR, REG, RFA, RIOC, RM, RNG, RYU, S, SALA, SANT, SAPS, SASK, SBT, SEL, SIU, SJRP, SMDB, SMF, SNM, SOM, SP, SRFA, SPF, SPSF, SQF, STL, STU, SVG, TAI, TAIF, TAMU, TAN, TEF, TENN, TEPB, TFC, TI, TKPM, TNS, TO, TU, UAM, UB, UCR, UEC, UFG, UFMT, UFP, UGDA, UJAT, ULM, UME, UNA, UNB, UNR, UNSL, UPCB, UPEI, UPNA, USAS, USJ, USM, USNC, USZ, UT, UTC, UTEP, UWO, V, VAL, VALD, VEN, VMSL, VT, W, WAG, WAT, WII, WELT, WFU, WMNH, WS, WTU, WU, Z, ZSS, ZT, CUVC, LZ, AAS, AFS, BHCB, CHAM, FM, PERTH, SAN.

References

Alagador, D. et al. 2016. Climate change, species range shifts and dispersal corridors: an evaluation of spatial conservation models. – Methods Ecol. Evol. 7: 853–866.

Anderson-Teixeira, K. J. et al. 2015. CTFS-ForestGEO: a worldwide network monitoring forests in an era of global change. – Global Change Biol. 21: 528–549.

Angelo, C. L. and Daehler, C. C. 2013. Upward expansion of fire-adapted grasses along a warming tropical elevation gradient. – Ecography 36: 551–559.

Araújo, M. B. and Guisan, A. 2006. Five (or so) challenges for species distribution modelling. – J. Biogeogr. 33: 1677–1688.

Araújo, M. B. et al. 2004. Would climate change drive species out of reserves? An assessment of existing reserve-selection methods. – Global Change Biol. 10: 1618–1626.

Araújo, M. B. et al. 2019. Standards for distribution models in biodiversity assessments. – Sci. Adv. 5: eaat4858.

Austin, K. G. et al. 2017. Trends in size of tropical deforestation events signal increasing dominance of industrial-scale drivers. – Environ. Res. Lett. 12: 054009.

Bagchi, R. et al. 2018. Forecasting potential routes for movement of endemic birds among important sites for biodiversity in the Albertine Rift under projected climate change. – Ecography 41: 401–413.

Baillie, J. and Zhang, Y.-P. 2018. Space for nature. – Science 361: 1051.

Bellard, C. et al. 2012. Impacts of climate change on the future of biodiversity. – Ecol. Lett. 15: 365–377.

Birdlife International. 2018. Endemic bird area spatial data. Q4

Brown, J. H. et al. 2003. Management of the semi-natural matrix. – In: Marquet, P. A. and Bradshaw, G. A. (eds), How landscapes change. Springer, pp. 327–343.

CBD Secretariat 2020. Zero draft of the post-2020 Global Biodiversity Framework, CBD/WG2020/2/3, Montreal, Canada. Q5

Ceballos, G. et al. 2015. Accelerated modern human-induced species losses: entering the sixth mass extinction. – Sci. Adv. 1: e1400253.

Chen, I.-C. et al. 2011. Rapid range shifts of species associated with high levels of climate warming. – Science 333: 1024–1026.

Corlett, R. T. and Tomlinson, K. W. 2020. Climate change and edaphic specialists: irresistible force meets immovable object? – Trends Ecol. Evol. in press. Q6

DeWalt, S. J. et al. 1999 Ethnobotany of the Tacana: quantitative inventories of two permanent plots of northwestern Bolivia. – Econ. Bot. 53: 237–260.

Di Marco, M. et al. 2015. Synergies and tradeoffs in achieving global biodiversity targets. – Conserv. Biol. 30: 189–195.

Dinerstein, E. et al. 2017. An ecoregion-based approach to protecting half the terrestrial realm. – BioScience 67: 534–545.

Dinerstein, E. et al. 2019. A global deal for nature: guiding principles, milestones, and targets. – Sci. Adv. 5: eaaw2869.

Diniz-Filho, J. A. F. et al. 2009. Partitioning and mapping uncertainties in ensembles of forecasts of species turnover under climate change. – Ecography 32: 897–906.

Dirzo, R. et al. 2014. Defaunation in the Anthropocene. – Science 345: 401–406.

Dudley, N. and Stolton, S. (eds) 2003. Running pure: the importance of forest protected areas to drinking water. – WWF and the World Bank, Gland, Switzerland and Washington DC.

Dudley, N. et al. 2018. The essential role of other effective area-based conservation measures in achieving big bold conservation targets. – Global Ecol. Conserv. 15: e00424.

Ehrlich, P. R. and Mooney, H. A. 1983. Extinction, substitution, and ecosystem services. – BioScience 33: 248–254.

Enquist, B. and Boyle, B. 2012. SALVIAS – the SALVIAS vegetation inventory database. – Biodivers. Ecol. 4: 288–288. Q7

Enquist, B. J. et al. 2016. Cyberinfrastructure for an integrated botanical information network to investigate the ecological impacts of global climate change on plant biodiversity. – PeerJ Preprints < https://peerj.com/preprints/2615.pdf>.

Enquist, B. J. et al. 2019. The commonness of rarity: global and future distribution of rarity across land plants. – Sci. Adv. 5: eaaz0414.

Fegraus, E. 2012. Tropical Ecology Assessment and Monitoring Network (TEAM Network). – Biodivers. Ecol. 4: 287–287.

Foden, W. et al. 2007. A changing climate is eroding the geographical range of the Namib Desert tree *Aloe* through population declines and dispersal lags. – Divers. Distrib. 13: 645–653.

Foden, W. B. et al. 2013. Identifying the world's most climate change vulnerable species: a systematic trait-based assessment of all birds, amphibians and corals. – PLoS One 8: e65427.

Forest Inventory and Analysis National Program. 2013. – <www.fia.fs.fed.us/>.

Garcia, R. A. et al. 2012. Exploring consensus in 21st century projections of climatically suitable areas for African vertebrates. – Global Change Biol. 18: 1253–1269.

Griscom, B. W. et al. 2017. Natural climate solutions. – Proc. Natl Acad. Sci. USA 114: 11645–11650.

Halpin, P. N. 1997. Global climate change and natural-area protection: management responses and research directions. – Ecol. Appl. 7: 828–843.

Hannah, L. et al. 2007. Protected area needs in a changing climate. – Front. Ecol. Environ. 5: 131–138.

Hannah, L. et al. 2014. 'Fine-grain modeling of species' response to climate change: holdouts, stepping-stones, and microrefugia.' – Trends Ecol. Evol. 29: 390–397.

Hannah, L. et al. 2019. Data from: 30% land conservation and climate action reduces tropical extinction risk by more than 50%. – Dryad Digital Repository, <http://dx.doi.org/10.5061/dryad.xxxxx>.

Heller, N. E. and Zavaleta, E. S. 2009. Biodiversity management in the face of climate change: a review of 22 years of recommendations. – Biol. Conserv. 142: 14–32.

Hengl, T. et al. 2017. SoilGrids250m: global gridded soil information based on machine learning. – PLoS One 12: e0169748.

Hijmans, R. J. et al. 2005. Very high resolution interpolated climate surfaces for global land areas. – Int. J. Climatol. 25: 1965–1978.

Hijmans, R. J. et al. 2017. Package 'dismo', ver. 1.1-4.

IPBES 2019. Summary for policymakers of the global assessment report on biodiversity and ecosystem services of the Intergovernmental Science-Policy Platform on Biodiversity and Ecosystem Services.

IUCN and UNEP-WCMC 2018. IUCN spatial data portal, accessed June 2018. – UNEP-WCMC, Cambridge, UK.

IUCN and UNEP-WCMC 2018. The world database on protected areas (WDPA) [On-line]. UNEP-WCMC, Cambridge, UK. <www.protectedplanet.net>, downloaded April 2018.

Johnston, A. et al. 2013. Observed and predicted effects of climate change on species abundance in protected areas. – Nat. Clim. Change 3: 1055–1061.

Kujala, H. et al. 2013. Conservation planning with uncertain climate change projections. – PLoS One 8: e53315.

Lenoir, J. et al. 2008. A significant upward shift in plant species optimum elevation during the 20th century. – Science 320: 1768–1771.

Loarie, S. R. et al. 2009. The velocity of climate change. – Nature 462: 1052–1055.

Maitner, B. S. et al. 2018. The bien r package: a tool to access the Botanical Information and Ecology Network (BIEN) database. – Methods Ecol. Evol. 9: 373–379.

Marquet, P. A. et al. 2019. Protected area management and climate change. – In: Lovejoy, T. and Hannah, L. (eds), Climate change and biodiversity. Yale Univ. Press, pp. 283–296.

Melillo, J. M. et al. 2016. Protected areas' role in climate-change mitigation. – Ambio 45: 133–145.

Merow, C. et al. 2013. A practical guide to MaxEnt for modeling species' distributions: what it does, and why inputs and settings matter. – Ecography 36: 1058–1069.

Merow, C. et al. 2014. What do we gain from simplicity versus complexity in species distribution models? – Ecography 37: 1267–1281.

Moilanen, A. 2007. Landscape zonation, benefit functions and target-based planning: unifying reserve selection strategies. – Biol. Conserv. 134: 571–579.

Moilanen, A. et al. 2005. Prioritizing multiple-use landscapes for conservation: methods for large multi-species planning problems. – Proc. R. Soc. B 272: 1885–1891.

Moilanen, A. et al. 2014. Zonation spatial conservation planning methods and software v.4, User manual, 288 pp.

Mulongoy, K. J. and Gidda, S. B. 2008. The value of nature: ecological, economic, cultural and social benefits of protected areas. – Secretariat of the Convention on Biological Diversity. Montreal, Canada, 30 pages.

Myers, N. et al. 2000. Biodiversity hotspots for conservation priorities. – Nature 403: 853–858.

Pecl, G. T. et al. 2017. Biodiversity redistribution under climate change: Impacts on ecosystems and human well-being. – Science 355: eaai9214.

Peet, R. K. et al. 2012. VegBank: a permanent, open-access archive for vegetation plot data. – Biodivers. Ecol. 4: 233–241.

Peters, R. L. and Darling, J. D. S. 1985. The Greenhouse Effect and Nature ReservesGlobal warming would diminish biological diversity by causing extinctions among reserve species. – BioScience 35: 707–717.

Phillips, S. J. et al. 2006. Maximum entropy modeling of species geographic distributions. – Ecol. Model. 190: 231–259.

Phillips, S. J. et al. 2008. Optimizing dispersal corridors for the cape proteaceae using network flow. – Ecol. Appl. 18: 1200–1211.

Rahbek, C. et al. 2019a. Building mountain biodiversity: geological and evolutionary processes. – Science 365: 1114–1119.

Rahbek, C. et al. 2019b. Humboldt's enigma: what causes global patterns of mountain biodiversity? – Science 365: 1108–1113.

Rockström, J. et al. 2009. Planetary boundaries: exploring the safe operating space for humanity. – Ecol. Soc. 14.

Sandel, B. et al. 2011. The influence of late quaternary climate-change velocity on species endemism. – Science 334: 660–664.

Serra-Diaz, J. M. et al. 2014. Bioclimatic velocity: the pace of species exposure to climate change. – Divers. Distrib. 20: 169–180.

The convention on biological diversity of 5 June 1992 (1760 U.N.T.S. 69)

Trabucco, A. and Zomer, R. 2019. Global aridity index and potential evapotranspiration (ET0) climate database v2. figshare. Dataset. – <https://doi.org/10.6084/m9.figshare.7504448.v3>.

Tuanmu, M.-N. and Jetz, W. 2014. A global 1-km consensus land-cover product for biodiversity and ecosystem modelling. – Global Ecol. Biogeogr. 23: 1031–1045.

Urban, M. C. 2015. Accelerating extinction risk from climate change. – Science 348: 571–573.

van Proosdij, A. S. J. et al. 2016. Minimum required number of specimen records to develop accurate species distribution models. – Ecography 39: 542–552.

Warren, R. et al. 2013. Quantifying the benefit of early climate change mitigation in avoiding biodiversity loss. – Nat. Clim. Change 3: 678–682.

Williams, P. et al. 2005. Planning for climate change: identifying minimum-dispersal corridors for the cape Proteaceae. – Conserv. Biol. 19: 1063–1074.

Wiser, S. K. et al. 2001. Managing biodiversity information: development of New Zealand's National Vegetation Survey databank. – N. Z. J. Ecol. 25: 1–17.

Author Queries

JOB NUMBER: 5166

JOURNAL: OIK_ECOG

Q1 IUCN and WCMC 2019 cited in the text but not provided in the list.
Q2 Enquist et al. 2012 cited in the text but not provided in the list.
Q3, Q8 Please insert doi-number for Dryad Digital Repository: <http://dx.doi.org/10.5061/dryad.xxxxx>.
Q4 Please provide the publisher name and location for the reference 'Birdlife International 2018'.
Q5, Q11 Please provide the publisher details.
Q6 Please update the reference 'Corlett and Tomlinson 2020'.
Q7 Enquist and Boyle 2012 provided in the list, but not cited in the text.
Q9 Hijmans et al., 2017 provided in the list, but not cited in the text.
Q10 IUCN and UNEP-WCMC 2018 provided in the list, but not cited in the text.
Q12 Please update the reference.
