# Supplementary material for: Protected areas network is not adequate to protect a critically endangered East Africa Chelonian: Modelling distribution of pancake tortoise, Malacochersus tornieri under current and future climates
Source: PLoS One. 2021 Jan 20;16(1):e0238669. doi: 10.1371/journal.pone.0238669 (PMC7816999; doi:10.1371/journal.pone.0238669)
Supplement: Attachment — Submitted filename: PLOS ONE Reviewer comments and responses.docx [file pone.0238669.s006.docx]

**PLOS ONE Revision: Responses to reviewer and editor comments**

**Manuscript ID: PONE-D-20-25666**

Revision of “Protected areas network is not adequate to protect a critically endangered East Africa Chelonian: Modelling distribution of pancake tortoise, *Malacochersus tornieri* under current and future climates”

Following the decision from the journal we made the following changes as per comment raised by the editor and reviewers.

**Comments and responses from the editor**

**Comment:** 1. Please ensure that your manuscript meets PLOS ONE's style requirements, including those for file naming. The PLOS ONE style templates can be found at

https://journals.plos.org/plosone/s/file?id=wjVg/PLOSOne_formatting_sample_main_body.pdf and

https://journals.plos.org/plosone/s/file?id=ba62/PLOSOne_formatting_sample_title_authors_affiliations.pdf

**Response**: Manuscript is formatted following PLOS ONE’s style requirement.

**Comment** 2. In your Methods section, please provide additional information regarding the permits you obtained for the work. Please ensure you have included the full name of the authority that approved the field site access and, if no permits were required, a brief statement explaining why.

**Response:** The full name of the authority that granted the permit and free access to field sites are provided in the method section.

**Comment:** 3. We note that you have indicated that data from this study are available upon request. PLOS only allows data to be available upon request if there are legal or ethical restrictions on sharing data publicly. For more information on unacceptable data access restrictions, please see http://journals.plos.org/plosone/s/data-availability#loc-unacceptable-data-access-restrictions.

In your revised cover letter, please address the following prompts:

a) If there are ethical or legal restrictions on sharing a de-identified data set, please explain them in detail (e.g., data contain potentially sensitive information, data are owned by a third-party organization, etc.) and who has imposed them (e.g., an ethics committee). Please also provide contact information for a data access committee, ethics committee, or other institutional body to which data requests may be sent.

b) If there are no restrictions, please upload the minimal anonymized data set necessary to replicate your study findings as either Supporting Information files or to a stable, public repository and provide us with the relevant URLs, DOIs, or accession numbers. For a list of acceptable repositories, please see http://journals.plos.org/plosone/s/data-availability#loc-recommended-repositories.

We will update your Data Availability statement on your behalf to reflect the information you provide.

**Response:** According to IUCN the pancake tortoise is critically endangered and threatened by international pet trade therefore the location data collected by authors is not publicly available. However, these data are available from the corresponding author upon reasonable request. Other secondary data used in this study are available from the original source as cited in the method section. We also added this information in our revised cover later

**Comment:** 4. We note that Figures 1-3 in your submission contain map images which may be copyrighted.

All PLOS content is published under the Creative Commons Attribution License (CC BY 4.0), which means that the manuscript, images, and Supporting Information files will be freely available online, and any third party is permitted to access, download, copy, distribute, and use these materials in any way, even commercially, with proper attribution. For these reasons, we cannot publish previously copyrighted maps or satellite images created using proprietary data, such as Google software (Google Maps, Street View, and Earth). For more information, see our copyright guidelines: http://journals.plos.org/plosone/s/licenses-and-copyright.

We require you to either (a) present written permission from the copyright holder to publish these figures specifically under the CC BY 4.0 license, or (b) remove the figures from your submission:

a. You may seek permission from the original copyright holder of Figures 1-3 to publish the content specifically under the CC BY 4.0 license.

We recommend that you contact the original copyright holder with the Content Permission Form (http://journals.plos.org/plosone/s/file?id=7c09/content-permission-form.pdf) and the following text:

“I request permission for the open-access journal PLOS ONE to publish XXX under the Creative Commons Attribution License (CCAL) CC BY 4.0 (http://creativecommons.org/licenses/by/4.0/). Please be aware that this license allows unrestricted use and distribution, even commercially, by third parties. Please reply and provide explicit written permission to publish XXX under a CC BY license and complete the attached form.”

Please upload the completed Content Permission Form or other proof of granted permissions as an "Other" file with your submission.

In the figure caption of the copyrighted figure, please include the following text: “Reprinted from [ref] under a CC BY license, with permission from [name of publisher], original copyright [original copyright year].”

b. If you are unable to obtain permission from the original copyright holder to publish these figures under the CC BY 4.0 license or if the copyright holder’s requirements are incompatible with the CC BY 4.0 license, please either i) remove the figure or ii) supply a replacement figure that complies with the CC BY 4.0 license. Please check copyright information on all replacement figures and update the figure caption with source information. If applicable, please specify in the figure caption text when a figure is similar but not identical to the original image and is therefore for illustrative purposes only.

The following resources for replacing copyrighted map figures may be helpful:

USGS National Map Viewer (public domain): http://viewer.nationalmap.gov/viewer/

The Gateway to Astronaut Photography of Earth (public domain): http://eol.jsc.nasa.gov/sseop/clickmap/

Maps at the CIA (public domain): https://www.cia.gov/library/publications/the-world-factbook/index.html and https://www.cia.gov/library/publications/cia-maps-publications/index.html

NASA Earth Observatory (public domain): http://earthobservatory.nasa.gov/

Landsat: http://landsat.visibleearth.nasa.gov/

USGS EROS (Earth Resources Observatory and Science (EROS) Center) (public domain): http://eros.usgs.gov/#

Natural Earth (public domain): http://www.naturalearthdata.com/

**Response:** We used the Natural Earth public domain data in all figures, and we mentioned it in the figure legend.

**5. Reviewers Comments to the Author and their responses**

Reviewer #1:

**Comment:** This paper investigates the suitability of future climate space for the critically endangered pancake tortoise, with a view to informing translocations and/or population management that will maximize sustainability of future populations in the face of climate change and other stressors (this species is harvested for the international pet trade). The study finds that similar to the current situation, most future suitable habitat is outside of protected areas. Further they identify additional sites where the species is not currently known to occur that could serve to bolster the population.

While this topic is a very important and the investigation a needed contribution in terms of critically endangered species management, there were some fundamental aspects that relate to species distribution modelling and species conservation that have not been covered/discussed. I have made a number of specific comments, but in general, think that the following need to be addressed to strengthen the paper:

**Response:** We appreciate the observations from the reviewer, and we have taken the comments raised on-board so as to improve the manuscript.

**Comment:** 1. More rigorous assessment of the climatic variables selected. How do these relate to the species’ life history and what mechanism of impact are they expected to have on population viability. Also, in terms of the model, what was the relationship with these variables and tortoise occurrence, and what was the relative effect size of each? Are there any potentially informative variables that were not included?

Related to the above, are there any other non-climatic variables for which information exist that might be added? The authors suggest that rock substrate is important. The authors also mention a previously published distribution model. How do the results of these models compare?

**Response:** We appreciate the reviewer for this observation. We provided response curves and variable importance to show how do the occurrence of the species is affected by the predictor variables. In addition, we added one paragraph at the start of discussion section to briefly explain how the climatic variables can affect pancake tortoise.

The most influential non-climatic variable that we thought could be important for our model was either pre-Cambrian rock layer or rocky outcrops/kopjes layer. However, we were unable to get those layers for our species distribution model (SDM) and we mentioned this layer in the study limitations as one of the layers which need to be considered in future studies. We also compared our findings with those of previous published model (see lines 399 – 403).

**Comment:** 2. The tables and figures are useful, but could their utility could definitely be enhanced further with the addition of details. For example, protected areas are not shown and in some instances country borders are also omitted. For the tables, it would be useful to compare current to predicted future situations.

**Response:** We thank the reviewer for this observation. We revised our figures and now they include country boundaries (all figures) and protected area polygons (in Fig. 2 and Fig. 3). For the Table 1 current and future climatic suitable habitats are shown with the percentage of the suitable area which overlaps with the climatic suitable area. In Table 2 we added the current suitable habitat and the percentage of protected area which overlaps with stable habitats.

**Comment:** 3. The extent to which the current distribution has been impacted by overharvesting is not clear. The current models do not highlight any suitable habitat in Zambia, but only one occurrence was included from the Zambian border. Is it likely that the species used to be more widespread in Zambia? If so, the discussion should rather conclude that lack of current records from the area preclude investigation of habitat suitability in Zambia

**Response:** It is true that we did not quantify overharvesting of the species in our species distribution model. This was due to the fact that we were unable to obtain such layer to be used in species distribution model and we mentioned this as one of layers which need to be considered in future studies. Also, it is true that our model did not predict any suitable habitat in Zambia, and we obtained two locations for pancake tortoises in Zambia from Chansa and Wagner (2006) with all of them located in Nakonde District which is along the border with Tanzania. We tried to contact those authors (Chansa and Wagner) and Zambia Wildlife Authority (ZAWA) for more pancake locations in Zambia without success and we did not find any other locations from the online databases. According to Chansa and Wagner (2006); Mwaya et al. (2018); Mwaya et al. (2019), pancake tortoises in Zambia occur only in Nakonde District that borders Tanzania however their existence is questionable because the population status reported by Chansa and Wagner (2006) as well as ZAWA (2000) is lower than the international exports for the species from Zambia (see Mwaya et al. 2018). Due to that, there is an argument that the Zambia population could be the results of smuggling/trafficking from other East African Countries especially Tanzania where species occurs naturally.

**Comment:** 4. Some more general discussion/framing of species management under climate change would be useful. There are so many species and large change is expected. Is moving species to far off locations really viable? How are these interventions prioritized? And by whom? The paper by Hannah (2020) details some of the most important corridors for conservation in Africa. There is definite overlap with the areas identified in this study and would be worth mentioning.

**Response:** We don’t think moving species far off locations is viable, but we suggested the translocation/introduction/re-introduction of the species to climatically suitable habitats. We revised and added one paragraph under Management implications section to explain prioritization of conservation interventions and who should be involved. We also consulted the provided reference while writing and revising this part, therefore we thank reviewer for this observation.

**Specific comments:**

**Comment:** Line 118 – there is no mention (later) of whether your current distribution compares well to this previous model. What the major findings and reasons for any potential differences?

**Response:** We appreciate the reviewer for this observation. We compared our results with that previous study in the discussion between lines 399 – 403.

**Comment:** Line 170, in what way would aquaculture threaten the tortoise? I assume this is a grouped categorization by the IUCN, but perhaps leave off the aquaculture part or include in inverted commas?

**Response:** Thanks for this observation, we decided to leave off the aquaculture part.

**Comment:** Lines 179-182 combine sentences to reduce repetition

**Response:** We appreciate this observation; the sentences were combined as suggested.

**Comment:** Fig. 1 Caption: I suggest reordering and rewriting along the following lines to make the buffer area clear from the outset: Current natural occurrence of pancake tortoise (*Malacochersus tornieri*), based on data obtained in this study, with a 1-degree wide buffer around each presence record, at (A) continental scale and (C) regional scale. (B) shows the Zambezian and Somalia biogeographical region.

**Response:** We thank for this caption; we adopted the caption with modification.

**Comment:** Line 233: is some measure of maximum temperature not important from a species perspective under climate change? How close to thermal maxima is the current study area likely to get? How do the variables selected compare to those used in previous studies on tortoises?

**Response:** We thank the reviewer for this observation. In our modelling procedure we included maximum temperature of the warmest month (BIO 5) but the variable was not selected due to collinearity. However, our modelling procedure selected mean temperature of the wettest quarter (BIO 8) as one of the temperature measures included during predictions. The exclusion of maximum temperature on modelling of reptile species including pancake tortoise was also observed in Bombi et al. (2013). On the other hand, two of our variables were similar to those of the previous study we mentioned (Bombi et al. 2013). Those variables were the mean temperature of the wettest quarter (BIO 8) and precipitation seasonality (BIO 15). We also added some text in the first paragraph of discussion to explain what the reason for variation on the selected variables could be.

**Comment:** Line 242-251 I am not quite sure that I understand the different algorithms – are you saying that you had 5 methods of accounting for pseudo-absences?

**Response:** We used a different method of accounting for pseudo-absences for each of the five algorithms (BIOCLIM, Multivariate Adaptive Regression Splines (MARS), Multiple Discriminant Analysis (MDA), Random Forest (RF) and Maximum Entropy (MaxEnt)), as proposed by Barbet-Massin et al. (2012). In other words, for each algorithm we used a proper pseudo-absence selection method designed and applied.

**Comment:** Section starting line 241 “Species distribution modelling”– it is not clear at which point the future projections are brought into the modelling. These are referred to as part of a general analysis in line 269, but there is no explicit separation of how current versus future estimates of range were produced.

**Response:** We thank the reviewer for this comment. We have addressed selection of future scenarios in the Bioclimatic Variables section. Together with that, we have also changed sentences between lines 241 and 244, where now it reads: "Selected models were projected into current and future scenarios and then binarized using the AUC threshold. This approach avoided the use of subjective thresholds. Ensembles from future scenarios were built as a committee average of binarized rasters“.

**Comment:** Line 290 – with regard to the potential habitat in Angola. Was this also found to be the case when pseudo-absences were only generated from within the boundary of the current range? See the description of how different selection of pseudo-absences influences model outcomes and utility in Merow et al. 2013. The selection of pseudoabsences should also be informed by the type of conservation intervention that is most feasible. For example, I would imagine that conservation interventions within the species current countries of occupancy would be more likely to succeed than attempts in countries far beyond where the species has ever occurred. Should the area in Angola really be considered as part of the conservation strategy? The species has no means to natural dispersal to reach the area in Angola, so any such intervention would need to be human mediated and require significant international collaboration. Is this feasible? If not, I would concentrate statistics on countries in the proximity of the species current range and leave additional areas as a passing mention (unless the politics of such species/climate change management is dealt with in more depth).

**Response:** We thank the reviewer for this contribution. We have not performed the pseudo-absence strategy mentioned, but we can say that our approach was indeed thought to comprise available areas for the species to be present as well as to improve predictions over each algorithm (as proposed by Barbet-Massin et al. 2012, which comprises a bigger set of algorithms, not just MaxEnt). Regarding areas for Angola, we choose to maintain it in the final manuscript to inform that, even with the total collapse of species habitats, by forward mentioned anthropogenic impacts, we could translocate the species to sustain its populations.

**Comment:** Having said this, what happens at the border of the Zambezian Somalia bioregion? Is the habitat entirely unsuitable? What do future models beyond this border suggest?

**Response:** We thank the reviewer for this comment. We did not consider the areas outside of Zambezian Somalia bioregion as the pancake tortoise distribution is restricted to this bioregion. Additionally, IUCN has mentioned that the species is non-migrant (see Mwaya et al. 2019) therefore it could be difficult to find the species naturally outside its current range. Therefore, our modelling strategy considered that species would remain within its bioregion; thus, we have not projected models beyond that limit. However, for future exploration it could be interesting to look at it and compare with areas where the species does occur naturally.

**Comment:** Table 1 – please indicate whether the area protected is the protected area that overlaps with the species distribution/potential distribution or whether it indicates the total area under protection in a particular country. I assume the former. It may also be useful to provide the protected area as a percentage. Combining the information in Tables 1 and 2 to indicate the proportion of PA that remains stable might also be useful?

**Response:** We thank the reviewer for this comment. It is true that the protected area presented in the Table 1 it is the one which overlaps with the species suitable habitat, and we indicated it in the table caption to conform with the reviewer comment. We accept the idea of presenting the protected area in percentage however, we hesitate to combine the information of Tables 1 and 2 to a single table as we thought the table will be very large.

**Comment:** Lines 319-333 and Tables. It is hard how to determine how the much larger numbers and percentages presented in lines 319-325 compare to the smaller percentages of stable protected area in lines 326-333. It might be better to concentrate the results on the area within protected areas currently suitable for these tortoises and how stable this remains over time, as well as loss and addition of potential new protected area that might become suitable?

**Response:** We revised the lines pointed out by the reviewer and we only presented the percentages and areas of suitable and stable habitats which fall under the current protected area network.

**Comment:** Table 2: It would also be useful to add information on the current situation to Table 2 for comparison purposes (unless these numbers are intended to sum to the current total)? Is there any protected area that was suitable that becomes entirely unsuitable?

**Response:** We appreciate the reviewer for this observation. We added the area of the current suitable habitat in the Table 2 as suggested. However, there is the variation between current suitable habitat and stable areas. This was due to the mechanism used to weigh the cells between the two maps whereby for those of stable areas weight is one, while cells in current suitable area weight is less than one. We also added this information in the figure caption. Our calculations of protected lands did focus on protected area network and not each protected area individually, therefore we not in a position to tell which suitable protected area becomes entirely unsuitable.

**Comment:** Line 312, Fig. 2 – it is hard to tell how suitable Zambia is without the inclusion of country borders on the map. Is the model working correctly if it does not predict currently suitable habitat in areas where observations have been made? What is so different about Zambian habitat that causes it not to be selected? Plotting part A and B of Figure 1 together might assist in identifying where the edge of the bioregion is in relation to the country borders.

**Response:** We appreciate the reviewer for this comment. On addressing it we added country boundaries in Fig 2. Regarding the prediction in Zambia, it is true that our model did not predict the suitable habitat in Zambia although we obtained two the occurrence points from the country. In the country, the species only exist in the northern part of Nakonde District that borders Tanzania (Chansa and Wagner 2006) but scientists have raised some concerns on this population, and they thought that it was a result of trafficking (Mwaya et al. 2018). Their argument is basing on population status reported by Chansa and Wagner (2006) and from Zambia Wildlife Authority (ZAWA) (2000) which were very low as compared to exports reported from CITES (Mwaya et al. 2018). We also merged Part A and B of Figure 1 however the edge which we were talking about is not from the bioregion. The edge which we mentioned was for the climatically suitable niche and thus has low climatic suitability, which, when applied at a threshold of the SDM, turns into an absence.

**Results: There are some key factors missing from the current results/discussion section:**

**Comment:** Why are the core drivers of the model not discussed? It would be useful to see response curves for individual variables, as well as effect sizes

**Response:** We appreciate the reviewer for this observation. We added response curves as supplementary information and added some explanation on the effects of variables to the species.

**Comment:** Related to the above, the discussion should include some mechanistic drivers of the potential patterns – why are the key variables the ones picked up by the analysis.

**Response:** We thank the reviewer for this comment. We explained in the methods (subsection Bioclimatic Variables) that variable selection was done by considering multicollinearity and some statistical artifacts. However, for the selected variables we added variable importance as supplementary file to show how each of the variables contributed in the model.

**Comment:** Are there any potential climate variables thought to influence the species breeding or foraging success that have not been included in the current models?

**Response:** We thank the reviewer for this observation. Due to cryptic nature of the species (Mwaya et al. 2019), studies which have clearly described the climatic variables that influence breeding and foraging success for the wild population of pancake tortoise are very limited. However, Loveridge and Williams (1957) noted that the species apparently emerges to feed only during the early morning hours. Additionally, Ewert et al. (2004) tried to describe how temperature influence sex determination on the hatchlings of the pancake tortoise in captivity, meaning that temperature is important factor for reproduction of the species. Therefore, using less correlated six bioclimatic variables (three for temperature and three for precipitation) was a better option to model the distribution of the pancake in its natural occurring areas, but we are open if you could point out the links for climatic variables which can be useful for our model.

**Comment:** Are soil data not available? It would be useful to include these data in the models if they are available.

**Response:** We appreciate the reviewer for this comment. It is true that adding the soil data can in one way or another improve model performance especially for species which are edaphic specialist (Corlett and Tomlinson 2020) such as plants (Bertrand et al. 2012). However, for our case we were unable to get any data which properly assigned the areas with the characteristics the species is found in (As we were not able to find any literature which has described pancake tortoise as the edaphic specialist). Despite that, we need to consider that this type of data (categorical) constraints projections from future climate scenarios, which may hide potential areas where the soil is not suitable, but climate is. This would also hide adaptation possibilities, once the species may adapt to different soil conditions easily.

Additionally, the soil data which we are aware of is the one from Soilgrids (Hengl et al. 2017) which has soil characteristics from the top to underground. Due to that we were unable to understand which soil layer could predict the presence of Precambrian rock/rocky outcrops/kopjes which are preferred habitats for pancake tortoise.

**Comment:** How does the representation of the current distribution compare to other models done for this species in the past.

**Response:** We compared our results with that previous study in the discussion. Please see line 399-403.

**Comment:** How stable does the area currently under protection remain?

**Response:** We thank the reviewer for this comment. The stability of the habitat which falls under the current protected area network is pointed out in lines 324-328**.**

**Comment:** To what extent is climate change really expected to add to the threat of the species?

**Response:** According to our model of which we used to predict the suitable habitat under climate change, we predicted that the species climatic suitable habitat will increase with the change in climate as shown in current and future climatic scenarios. This can be seen in Fig. 2 as well as in Table 1. However, the major threat we predicted is that most of the suitable habitats will continue to lie outside of protected area network and these areas will expand with climatic changes.

**Comment:** It appears that conservation of the species outside of the protected area is currently a priority regardless of climate change, but the extent to which climate change may change this in future is what needs to be discussed. Is there anywhere in particular, considering land-use and potential for other threats, where you would recommend the establishment of new protected areas to protect this species? [This would be in areas least impacted by climate change and most suitable for the species].

**Response:** As we mentioned in the discussion that the current and future suitable habitats for the species are lying outside the current protected area network. Therefore, considering land use changes in the area which are not protected, the species will be threatened mostly in areas outside of the current protected area network. Therefore, expanding the current protected area network to cover the current suitable habitats is highly recommended as species is highly threatened with international live animal trade. This is what we recommended in the manuscript.

**Comment:** For the reported suitable ranges (lines 290-300), how was ‘suitable area’ quantified? I understand from the methods, there was a committee averaging technique applied, but this resulted in cases where some of the colours represent almost no agreement of presence. Which degree of consensus of suitable area (or colour on the map) was classified as ‘suitable’ for the numbers reported in the Tables?

**Response:** We thank the reviewer for this comment. As we addressed in lines 248-253, instead of using a threshold to consider if the species was absent or present we used a weighted method, where, e.g. if a cell had 0.5 value (i.e. 50% chance of occurring), we would sum just 50% of the cell area into the total area occupied by the species. The same being applied in each cell of the study area. We also added the provided example in the manuscript.

**Comment:** Fig. 2 There does not appear to be a large difference between the RCPs in terms of high match areas (>0.5; yellow to red). Why might this be?

**Response:** We appreciate the reviewer for this observation. The minor changes between RCPs is probably being influenced by bio3 - isothermality - which is very strong in the tropics and very strong in our models. This variable represents how temperature changes along the year. In other words, how different winter and summer temperatures are from each other. In the tropics, the difference between those seasons is almost non-existent and this will probably not change with climate change, considering it is an effect that is caused by sun position along the year (in solstice, sun is equally apart from equator no matter if it is summer or winter). Summed to that, we can see that this red area is almost in equator, where the effect described is very intense.

**Comment:** Fig. 3 please indicate the location of protected areas on this map

**Response:** Protected area polygons were added on the mentioned figure.

**References mentioned on first reviewer’s comments and responses**

Bertrand, R., Perez, V. and Gégout, J.‐C. (2012), Disregarding the edaphic dimension in species distribution models leads to the omission of crucial spatial information under climate change: the case of Quercus pubescens in France. Glob Change Biol, 18: 2648-2660. doi:10.1111/j.1365-2486.2012.02679.x

Barbet-Massin M, Jiguet F, Albert CH, Thuiller W. (2012). Selecting pseudo-absences for species distribution models: How, where and how many? Methods Ecol. Evol.;3: 327–338. doi:10.1111/j.2041-210X.2011.00172.x

ZAWA (Zambia Wildlife Authority) 2000. A Survey Report on Zambia as a Range State for the Pancake Tortoise (*Malacochersus tornieri*). Annual report to CITES. 5 pp. https://cites.org/common/cop/13/inf/E13i-04.pdf.

Mwaya, R.T., Moll, D., Malonza, P.K. & Ngwava, J.M. (2018). *Malacochersus tornieri* (Siebenrock 1903) – Pancake Tortoise, Tornier’s Tortoise, Soft-shelled Tortoise, Crevice Tortoise, Kobe Ya Mawe, Kobe Kama Chapati. In: Rhodin, A.G.J., Iverson, J.B., van Dijk, P.P., Stanford, C.B., Goode, E.V., Buhlmann, K.A., Pritchard, P.C.H. & Mittermeier, R.A. (Eds.). Conservation Biology of Freshwater Turtles and Tortoises: A Compilation Project of the IUCN/SSC Tortoise and Freshwater Turtle Specialist Group. Chelonian Research Monographs.

Chansa, W. & Wagner, P. (2006). On the status of *Malacochersus tornieri* (Siebenrock, 513 1903) in Zambia. Salamandra Bonn. 2006;42: 187.

Mwaya, R.T., Malonza, P.K., Ngwava, J.M., Moll, D., Schmidt, F.A.C. & Rhodin, A.G.J. 2019. *Malacochersus tornieri*. The IUCN Red List of Threatened Species 2019: e.T12696A508210. http://dx.doi.org/10.2305/IUCN.UK.2019-1.RLTS.T12696A508210.en

Ewert, M.A., Hatcher, R.E & Goode, J.M. (2004). Sex Determination and Ontogeny in *Malacochersus tornieri*, the Pancake Tortoise. Journal of Herpetology, 38(2): 291-295. https://www.jstor.org/stable/1566229

Loveridge, A. & Williams, E.E. 1957. Revision of the African tortoises and turtles of the suborder Cryptodira. Bulletin of the Museum of Comparative Zoology Harvard College 115(6): 160-557 + 18 pl.

Corlett, R. T. and Tomlinson, K. W. 2020. Climate change and edaphic specialists: irresistible force meets immovable object? Trends Ecol. Evol. 35(4):367-376. doi: 10.1016/j.tree.2019.12.007.

Merow, C., Smith, M.J. & Silander, J.A. (2013) A practical guide to MaxEnt for modeling species’ distributions: what it does, and why inputs and settings matter. Ecography, 36, 1058-1069.

Hannah, L., Roehrdanz, P.R., Marquet, P.A., Enquist, B.J., Midgley, G., Foden, W., . . . Svenning, J.C. (2020) 30% land conservation and climate action reduces tropical extinction risk by more than 50%. Ecography.

Bombi P, D’Amen M, Luiselli L. From Continental Priorities to Local Conservation: A Multi-Level Analysis for African Tortoises. PLoS One. 2013;8: 1–9. doi:10.1371/journal.pone.0077093

Hengl T, Mendes de Jesus J, Heuvelink GBM, Ruiperez Gonzalez M, Kilibarda M, Blagotić A, et al. (2017) SoilGrids250m: Global gridded soil information based on machine learning. PLoS ONE 12(2): e0169748. https://doi.org/10.1371/journal.pone.0169748

**Reviewer #2: General comments and responses:**

**Comment:** This paper examines the distribution of critically endangered pancake tortoises. The authors used occurrences obtained from Tanzania, Kenya and Zambia to estimate suitable and stable areas in other countries (projecting/predicting). They also used a protected area network to assess how many of the suitable and stable areas are protected for the conservation. The authors conclude most of the identified suitable habitats remain outside of protected areas. I think this is a sound contribution to the literature. I have provided specific comments below, aimed at clarification of methods and results. In addition, particularly in the discussion, I have suggested ways the text can be streamlined to flow better and be more concise and direct. Finally, I think some added text addressing some of the problematic areas of SDM at the end of the discussion might make this paper stronger. Specific comments are below, but in general I am referring to how SDMs are indeed frequently used, but they are also very controversial. So I just suggest you consider briefly addressing this and indicate how your study deals with these issues (e.g., by careful selection of data points etc.).

**Response:** We thank the reviewer for the comments and suggestions, we addressed them during our revision. We also added some text in the ‘Conclusion and study limitations, on limitations of SDMs.

**Specific comments and responses:**

**Comment:** Line 43: It seems like a few more citations might be helpful to demonstrate the growing interest in species distribution modeling.

**Response:** We added some few more citations as suggested.

**Comment:** Line 53: Additional citations seem warranted here.

**Response:** We added more citations as suggested.

**Comment:** Line 54: Provide citation, if possible.

**Response**: Citations were added as suggested

**Comment:** Line 81: Consider starting the sentence differently because the previous sentence also beings with “although”.

**Response:** The sentence has been revised and now it starts with the words ‘Despite the fact that…’

**Comment:** Line 86: It would be more concise to say “…has indicated climate-matching of recipient sites is important for understanding suitability of these areas for introduced/translocated species”, or something similar. In general, this sentence is a bit cumbersome and seems to be discussing translocation, so you might want to set that up before this sentence with a better transition. Maybe just lead in with something simple transitioning from “no study has assessed climate change in these tortoises” to why re-introductions might be necessary.

**Response:** We thank the reviewer for this suggestion. We revised the mentioned part and lines as pointed out by the reviewer.

**Comment:** Line 93: I think this phrasing is a little problematic “because SDM is the most widely accepted method of predicting climatically suitable habitats”. I might eliminate this part. SDM is frequently used, true, but it is also very controversial, and refers to many different methods, including occupancy modeling. So SDM ends up being an umbrella term for several of different ways of mapping species distribution.

**Response:** We thank the reviewer for this suggestion, we revised the sentence and remove the part pointed out by the reviewer.

**Comment:** Line 97: What is the implication for “there are endangered species that inhabit areas outside of protected lands”? I think this information will help make a stronger point. In essence—what is the point you are trying to make? I would suggest moving Lines 102-104 to the beginning of the paragraph, so the main point is clearer from the beginning. Only slight sentence re-structuring would be needed for the rest of the paragraph if you re-arranged this way.

**Response:** We placed the mentioned sentence at the beginning of the paragraph and revise the paragraphs so as to make it clearer.

**Comment:** Line 112: See earlier comment and consider removing “SDMs are essential”. This part is unnecessary. What is essential is the examination of how well protected areas perform, not the SDM. Plenty of other methods can be used to achieve the same goal.

**Response:** We thank the reviewer for this comment. We revised the mentioned sentence and it now reads ‘SDMs can be used’ instead of ‘SDMs are essential’**.**

**Comment:** Lines 113-114: Correct, and there is a lot of controversy over SDM’s, for example, in the data used to generate them (see below). I am not saying you need to reference these papers, I’m just illustrating a point that drastically different results occur with even small differences in data inputs. I wonder if controversy should be addressed here, or perhaps in the discussion in a way that shows you have thought about this and carefully selected your data and approach.

Rodda, G. H., C. S. Jarnevich, and R. N. Reed. 2009. What parts of the US mainland are climatically suitable for invasive alien pythons spreading from Everglades National Park? Biological Invasions 11:241–252.

Pyron, R. A., F. T. Burbrink, and T. J. Guiher. 2008. Claims of Potential Expansion throughout the U.S. by Invasive Python Species Are Contradicted by Ecological Niche Models. PLoS ONE 3:e2931

Rodda, G. H., C. S. Jarnevich, and R. N. Reed. 2011. Challenges in Identifying Sites Climatically Matched to the Native Ranges of Animal Invaders. PLoS ONE 6:e14670.

**Response:** We appreciate the reviewer for this observation. In our SDM we used five algorithms with each algorithm having different pseudo-absence selection as suggested by Barbet-Massin et al. 2012 in order to reduce inconsistences. In addition, we mentioned the limitation of our study in the section titles ‘Conclusion and study limitations’.

Barbet-Massin M, Jiguet F, Albert CH, Thuiller W. 2012. Selecting pseudo-absences for species distribution models: How, where and how many? Methods Ecology and Evolution, 3: 327–338. doi:10.1111/j.2041-210X.2011.00172.x

**Comment:** Line 125: “the occurrence of more stable areas over time” seems like it needs to be more explicitly defined, but hopefully this will be more clear in the methods.

**Response:** We thank the reviewer for this observation, we revised the sentence and it now reads ‘the occurrence of potential refuge areas for the species over time’.

**Comment:** Line 127: Consider re-phrasing as “This study may inform specie management approaches, including identifying suitable areas for translocation and the establishment of nature reserves where species can be protected with minimal human intervention [10]. This way is more concise (the sentence is long). But more importantly, the way you have this written reads as a promotion for SDM’s and I don’t think that is warranted without more information on how they need to be used carefully. The discussion probably needs this information.

**Response:** We appreciate this comment from the reviewer. We considered the statement and adopted as suggested.

**Comment:** Line 159: Remove “up”

**Response:** The word ‘up’ was removed as suggested.

**Comment:** Lines 160-162: “…tortoises are disjointly distributed from the northern to southern areas, passing though the central to south-eastern regions of the country”. This reads awkwardly and is a bit confusing.

**Response:** The sentence has been revised and it now reads ‘…the distribution of pancake tortoises is disconnected from the northern to southern areas, lying between central to south-eastern regions of the country’.

**Comment:** Line 175: Remove “on the ground”

**Response:** We removed the words as the reviewer suggested.

**Comment:** Lines 175-178: Can you reference one of your maps for where your field sites were, generally? Or is this best left unsaid because of poaching concerns? I mention this because many will not know where these areas are without a visual.

**Response:** We appreciate the reviewer for this observation. However, as pancake tortoise is critically endangered and the animal is at high risk of illegal offtake, we thought it could be better to leave the surveyed sites unsaid. This is just for ethical considerations; however, we are open to share these locations upon reasonable request.

**Comment:** Lines 185-187: I like that you specified how you cleaned the records and would say that as much information here as possible is warranted, because even one incorrect data point can throw off model results.

**Response:** We appreciate for this support.

**Comment:** Lines 188-190: It is not clear why you would need elevation data to get coordinates. Please clarify. Further, is this a source of error? These are extrapolated data and could inflate errors in your predictions. Again, I think some careful explanation of how these models are sensitive to user inputs, and how you addressed this carefully in your approach is important.

**Response:** We appreciate the reviewer for this comment. We did not extract elevation data from Elevation Map (<https://elevationmap.net/>) website. We only used the website to obtain coordinates of locations which we were interested especially those from the hills as it easy to spot them by looking at contours. After we obtained the coordinates, we plotted them by using GIS and send the map to experts who are current/previous work in the area for confirmation. We do all of this so as to be more precise with the location.

**Comment:** Lines 216-219: Provide a citation indicating they are more realistic.

**Response:** We thank the reviewer for this comment. We revised the pointed lines and add citation as suggested.

**Comment:** Line 214: What is a Representative concentration pathway?

**Response:** RCPs are the scenarios built by Intergovernmental Panel on Climate Change (IPCC) to guide research regarding future possibilities for our climate. Each RCP represents a different rate of greenhouse gas emission. For studies which involved species distribution models these terms are normally used however we added citations in the manuscript so that if a reader will need more details can look for them.

**Comment:** Line 219: Variations in future scenarios? Please consider adding a little more detail.

**Response:** We thank the reviewer for this comment. We rephrased the sentence and it now reads "Future scenarios' uncertainty were also accessed through ten ...".

**Comment:** Line 220: Are you saying you ran 10 models to quantify variation? Please clarify.

**Response:** Each GCM is a model trying to explain how the atmosphere works. When we use multiple GCMs, we are dissolving the effect of one unique GCM and improving our predictions (Oh and Suh, 2017). We also added this text in the manuscript.

Oh, S., Suh, M. (2017). Comparison of projection skills of deterministic ensemble methods using pseudo-simulation data generated from multivariate Gaussian distribution. Theoretical and Applied Climatology 129, 243–262. https://doi.org/10.1007/s00704-016-1782-1

**Comment:** Lines 222-223: What are all these acronyms? Define them as models and list out what they stand for or provide a citation that identifies them.

**Response:** The acronyms are the name of the institutions that built those models followed by the model name and version. We thought that explaining them in the text will not contribute to understanding the methods. In this way, we added a supplementary table which defines those acronyms.

**Comment:** Line 225: What is BIO 18? Bio 19? Clarify.

**Response:** These are among 19 bioclimatic variables of which BIO 18 means precipitation of the warmest quarter while BIO 19 means precipitation of the coldest quarter as explained in the text.

**Comment:** Lines 224-230 are confusing. What are you doing? Perhaps say something like “To evaluate different models we first eliminated x, because of …., and then eliminated y, to assess …..” Or some similar phrasing.

**Response:** We thank the reviewer for this comment. We first thought that this could help readers to understand what are those statistical artifacts, but as it is causing more confusion than clarification, we suppressed the sentence from lines 210 to 213 ("Those artifacts are generated due to a difference in which quarter is the warmest (e.g. BIO18), causing the precipitation of one cell to be the sum from January-February-March, while the very next cell is the summed precipitation from February-March-April").

**Comment:** Line 252: What standard procedures?

**Response:** We thank the reviewer to point this out. We rephrase it to: ‘Algorithms were implemented using standard parameterization from the sdm package [53] hence the algorithms were not tuned.’

**Comment:** Line 261: How does this avoid subjective thresholds? Perhaps re-phrase as “The selected models were binarized using the AUC threshold to avoid use of a subjective threshold”. Include citation?

**Response:** We thank the reviewer comment and we re-phrased the sentence ant it now reads as ‘Selected models were projected into current and future scenarios and then binarized using the AUC threshold to avoid the use of subjective thresholds.’

**Comment:** Line 272: Consider removing “furthermore”.

**Response:** The word ‘furthermore’ was removed in the sentence.

**Comment:** Line 284: You are letting R average the models built with different algorithms, which is fine. I just wonder if you can extract the information and depict a table of each algorithm’s relative performance. This is just a suggestion.

**Response:** We appreciate this suggestion however this table has 200 rows. In this way we opted not to include it.

**Comment:** Line 300-301: You say “will continue to” and “will expand” etc. Clarify that you are predicting these things with “we predict that…” for example. These results are just predictions, not an indication of what will happen with 100% certainty. Fix this throughout the Results where applicable. E.g., Lines 320, 323, 331

**Response:** We appreciate the reviewer for this observation, we corrected the sentences accordingly and we revised the result and discussion sections in order to make further corrections.

**Comment:** Line 329: Define stability in this context; likewise define in Table caption on Line 341. How did you determine stability?

**Response:** We thank the reviewer for this comment. We have changed Table caption as reads: ‘Potential climatic stable areas/habitats (in km2) for the pancake tortoise per each country present in the Zambezian and Somalia biogeographical regions. Climatic stability was inferred by extracting all values greater than zero from the raster and obtaining the 90th, 95th and 99th quantiles, returning low, mid and high stability, respectively. Area was calculated with a suitability weighted approach. …’ To address the definition of stability, we rewrote our second objective, where now it reads: "(ii) the occurrence of climatically persistent areas over time (henceforward, stable areas) ...".

**Comment:** Line 335: Is ‘red sheds’ a typo?

**Response:** We thank for this observation; we removed the word ‘red sheds’ in the sentence.

**Comment:** Line 345: Begin with “We predicted that…” rather than “Our SDM predicted”

**Response:** Corrected as suggested.

**Comment:** Line 359: Reads awkwardly. What do you mean by “the isolation of pancake tortoise populations is also occurring within Tanzania…”? Does this refer to future predictions? Should you re-phrase as “Based on our model predictions we expect tortoise populations to become isolated…”?

**Response:** Thank you for this observation. The sentence mentioned is not from our findings, but we were trying to show that even in the naturally occurring populations of Tanzania and Kenya the isolation is still existing. This was added as in our results Angola was isolated in the far west of the region. As the reviewer pointed out that the sentence reads awkwardly, we revised it and it now reads as ‘This scenario of isolation of pancake tortoise populations has been also recorded in Tanzania [42] and Kenya [10], where the species exists naturally’

**Comment:** Line 361: Re-phrase, reads awkwardly.

**Response:** We revised the sentence and it now reads ‘The absence of pancake tortoise in the countries which we predicted to have the climatically suitable habitats could be due to their behaviour of being non-migrant [10–12]’

**Comment:** Line 363: “Furthermore” is not necessary and reads a little awkwardly in this context.

**Response:** We removed the word ‘Furthermore’ in the mentioned sentence and replaced it with the word ‘On the other hand’.

**Comment:** Lines 356-370 could use some tightening up; the flow if the text is a bit choppy and could be streamlined a bit. Instead of “As pancake tortoises prefer areas featuring Precambrian rocks, the presence of other rock types between the suitable habitats could act as a distribution barrier [13]. Therefore, this could be another reason for the non-existence of pancake tortoises in some climatically suitable habitats; however, the species may occur in the Zambezian floral region, provided that suitable habitat is available” try “Pancake tortoises do not occur in some climatically suitable habitats (citation/Fig?). The prevalence of non-Precambrian rock types between areas where we predict suitable habitat may preclude occupancy by Pancake tortoises, as they prefer areas dominated by Precambrian rocks [13].”

**Response:** We appreciate the reviewer for this suggestion. We adopted the text as suggested.

**Comment:** Line 370: This is confusing “however, the species may occur in the Zambezian floral region, provided that suitable habitat is available [13,37]” given the previous statement saying that they often don’t occur in suitable habitat.

**Response:** We thank the reviewer for this observation. The mentioned text was dropped the during revision.

**Comment:** Lines 371-383: As with the previous comment this text could be re-written to be more concise and to flow better.

**Response:** We thank the reviewer for this observation. We revised this part and we think it is now clearer than before.

**Comment:** Line 387: Remove “Additionally”. Also, as above, this sentence could use some restructuring for conciseness and clarity. For example, I might re-phrase as follows: “We recommend robust habitat suitability studies in these countries and further quantification of occupancy status given the species apparently occupies suitable habitats in the nearby Zambezian and Somalian biogeographical regions [37],[13].”

**Response:** We the reviewer for this comment and we adopted the text as suggested.

**Comment:** Line 391-393: Consider re-phrasing as “Protected areas are critical tools for biodiversity conservation [23], yet the African Protected Areas Network offers inconsistent protection to tortoise species [33].

**Response:** We considered the text provided by the reviewer and rephrased our text as suggested.

**Comment:** Line 402: “none of the recorded species is within” reads awkwardly. Consider re-phrasing as “while in Zambia, the species does not occur within the Protected Area Network [12,13].”

**Response:** The sentence was revised as per reviewer suggestion.

**Comment:** Line 403: Consider re-phrasing as “In Tanzania, only 4 out of 22 national parks are occupied by pancake tortoises.” Also, is this based on your results? If so, clarify with something like “we predict that in Tanzania only 4 of 22 parks….”

**Response:** This argument was based on occurrence points we collected in Tanzania. Therefore, the statement now reads as ‘Based on occurrence points we collected, in Tanzania, only four out of 22 national parks are occupied by pancake tortoises’

**Comment:** Lines 404-409: Consider re-phrasing for better flow: “The pancake tortoise’s suitable habitat is largely unprotected in both the current and the future scenarios, likely increasing the risk of over exploitation and exacerbating negative effects of habitat destruction as in Tanzania [18], Kenya [10,12] and Zambia [12,13]. Likewise, ectoparasite prevalence is higher outside of protected area [59], potentially increasing risk to the species”.

**Response:** We thank the reviewer for this paraphrasing suggestion, we adopted the text as commented.

**Comment:** Line 412: Consider re-phrasing as “Because the range of Pancake tortoises does not include current and future predictions for climatically suitable habitats, we recommend future studies be conducted in areas where pancake tortoises do not exist to confirm absence of the species.”

**Response:** We thank the reviewer for this suggestion. We adopted the text with some modifications and it now reads as ‘Because the current natural range for pancake tortoise does not include some of our current and future predictions for climatically suitable habitats, we recommend future studies be conducted in areas where pancake tortoises do not exist to confirm the absence of the species.’

**Comment**: Line 444: “or even their inversion into a trend of growth.” This part seems a bit speculative at this point. I would consider removing, but if you keep, perhaps consider re-phrasing this sentence with something similar to: “We predict expansion of suitable habitat for pancake tortoises in the future, which may conserve populations of this critically endangered reptile”.

**Response:** We appreciate the reviewer for this observation. We decided to keep the sentence by adopting the provided text.

**Comment:** Line 444-448: This is an important sentence. I would re-phrase as “Importantly, the largest proportion of suitable habitat is outside of the current Protected Area Network, therefore we suggest the Pancake tortoise be upgraded in its listing status from CITES Appendix II to Appendix I.” This is more streamlined and direct.

**Response:** We thank the reviewer for this comment. We adopted the text as suggested.

**Comment:** Line 449-452: Re-phrase as “Because our results were largely based on use of climactic variables, our findings should not be treated as ready-made for on-the-ground application but could be used as one of many tools to help in conservation planning of Pancake tortoises.” This is a bit more streamlined and concise.

**Response:** The sentence was re-phrased as suggested by the reviewer.

**Comment:** Line 453: Re-phrase as “Our decision to use primarily climate variables was because climate variables drive most of the specie’s distribution”. This is a lot more concise, or less repetitive.

**Response:** We appreciate the reviewer for this suggestion. We adopted the text with some modification and therefore in now reads as ‘Our decision to use primarily climate variables was because they drive most of the species’ distribution [60].’

**Comment:** Line 462: I think a stronger ending is warranted. I might suggest saying “Our study provides a solid foundation for future development of conservation measures aimed at protecting populations of the critically endangered pancake tortoise.” or something similar.

**Response:** We thank the reviewer for the suggestion, we adopted as it is.
